# A Review of Online Evidence-based Practice Point-of-Care Information Summary Providers

**DOI:** 10.2196/jmir.1288

**Published:** 2010-07-07

**Authors:** Rita Banzi, Alessandro Liberati, Ivan Moschetti, Ludovica Tagliabue, Lorenzo Moja

**Affiliations:** ^3^University of MilanMilanItaly; ^2^University of Modena and Reggio EmiliaModenaItaly; ^1^Italian Cochrane CentreMario Negri Institute for Pharmacological ResearchMilanItaly

**Keywords:** Point-of-care system, Internet information, evidence-based practice, evidence-based medicine, information science

## Abstract

**Background:**

Busy clinicians need easy access to evidence-based information to inform their clinical practice. Publishers and organizations have designed specific tools to meet doctors’ needs at the point of care.

**Objective:**

The aim of this study was to describe online point-of-care summaries and evaluate their breadth, content development, and editorial policy against their claims of being “evidence-based.”

**Methods:**

We searched Medline, Google, librarian association websites, and information conference proceedings from January to December 2008. We included English Web-based point-of-care summaries designed to deliver predigested, rapidly accessible, comprehensive, periodically updated, evidence-based information to clinicians. Two investigators independently extracted data on the general characteristics and content presentation of summaries. We assessed and ranked point-of-care products according to: (1) coverage (volume) of medical conditions, (2) editorial quality, and (3) evidence-based methodology. We explored how these factors were associated.

**Results:**

We retrieved 30 eligible summaries. Of these products, 18 met our inclusion criteria and were qualitatively described, and 16 provided sufficient data for quantitative evaluation. The median volume of medical conditions covered was 80.6% (interquartile range, 68.9% - 84.2%) and varied for the different products. Similarly, differences emerged for editorial policy (median 8.0, interquartile range 5.8 - 10.3) and evidence-based methodology scores (median 10.0, interquartile range 1.0 - 12.8) on a 15-point scale. None of these dimensions turned out to be significantly associated with the other dimensions (editorial quality and volume, Spearman rank correlation *r* = -0.001, *P* = .99; evidence-based methodology and volume, *r* = -0.19, *P* = .48; editorial and evidence-based methodology, *r* = 0.43, *P* =.09).

**Conclusions:**

Publishers are moving to develop point-of-care summary products. Some of these have better profiles than others, and there is room for improved reporting of the strengths and weaknesses of these products.

## Introduction

In 1996, Richard Smith sought to identify the main characteristics that medical information sources developed over the next decade should have to guide doctors in their practice [1,2]. He concluded that these tools should be able to answer complex questions, be connected to a large, valid database, and be electronic. Today, busy clinicians have access not only to Medline, but to many online information solutions that are now faster, have a broader and deeper reach into the plethora of medical literature, and can quickly provide current information directly related to their everyday practice. This approach, supported by advances in the technical areas of powerful real-time information systems, fits well with medical information consumed when patients and practitioners interact at the so-called point of care, which requires information formatted differently than traditional information sources, such as textbooks [3].

**Figure 1:**
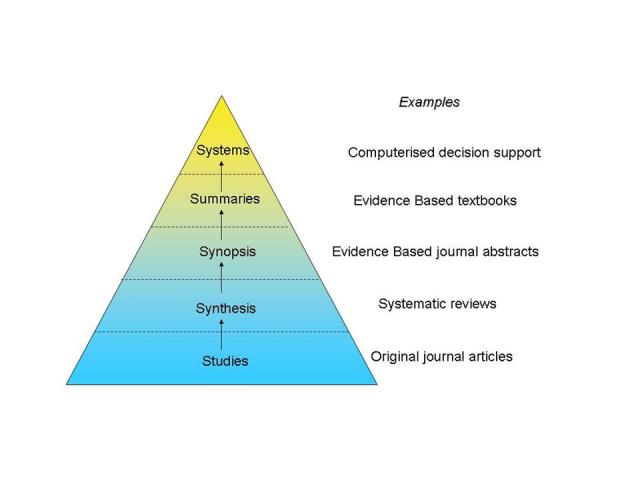
The ‘‘5S’’ levels of organization of evidence from health care research (adapted from Haynes [6])

The unquestionable advantage of online point-of-care tools is that they facilitate the selection and summary of research findings and provide friendly interfaces to improve retrieval, synthesis, organization, and application of this information [4]. The model within evidence-based practice (EBP) information summaries was first described is the “5S” paradigm, which provides guidance for using the most “evolved” information services when searching for the best current evidence [5,6]. This model guides those seeking information related to a clinical problem to begin their search at the highest level resource available, such as comprehensive and sophisticated information tools (ie, systems and summaries). Lower level resources, such as systematic assemblies of the evidence (ie, synthesis and synopsis) and individual studies, should only be searched when there is no evidence-based information system available (Figure 1) [5,6].

In the context of the 5S paradigm, summaries have been described as having a pivotal role as they can integrate the best available evidence from lower layers (drawing on studies and synthesis) to provide information on management options for a given health condition. Summaries are also the basis on which more interactive computerized decision support tools, or “systems,” are usually developed [6].

Most online summaries are promoted as “evidence-based” [7] implying that their contents are developed through a periodic and systematic search and critical evaluation of medical literature. The claim of being “the most authoritative and accessible point-of-care medical reference available to physicians and other health care professionals on the Internet” is just one example of the emphatic marketing claims used for product advertising [8]. However, criteria for selecting clinically important evidence are not always explicit, raising questions about the quality of information [9]. As online EBP point-of-care summary providers are mushrooming and a substantial a priori trust by clinicians is to be expected, it is of prime importance to assess the relevance and validity of point-of-care summaries, particularly in terms of quality of the content and comprehensiveness.

The objective of this study was to describe online EBP point-of-care summary providers and to evaluate their content and editorial policy against their claims of being “evidence-based.” As for all research, the quality of point-of-care summaries needs to be evaluated to ensure the real usefulness of these products for clinical practitioners. We arbitrarily postulated that coverage of medical knowledge, editorial policy, and content quality (three desirable criteria) would have been among the properties of the best products, being fully aware that basing our evaluation on these criteria would constitute a content-centred evaluation rather than a user-centered or experience/satisfaction evaluation.

## Methods

### Eligible EBP Point-of-Care Summary Providers

This study focused on providers of EBP point-of-care summaries, which can be broadly defined as web-based medical compendia specifically designed to deliver predigested, rapidly accessible, comprehensive, periodically updated, and evidence-based information (and possibly also guidance) to clinicians (see Table 1 for definitions). Thus, in order to be included in our analysis, a product had to be an online-delivered tertiary publication (summary) that is regularly updated, claims to provide evidence-based information to physicians and other health professionals, and is to be used at the bedside. As previously stated, the term “point of care” indicates the point where patients and practitioners interact, particularly referring to the context of the provider-patient dyad. Here, “point of care” applies to a summarized reference content describing alternative options in clinical practice, rather than technical solutions optimized for the use at the bedside. We restricted our analysis to summaries published in English as the primary language.

The following online information resources were excluded (see Table 1 for definitions): (1) guideline databases (as they are intended to provide recommendations rather than information); (2) medical meta-lists and search engines, both medicine-specific and general (as they point the user toward the right place to find information rather than providing information themselves) [10]; (3) literature surveillance alerting systems (as they monitor a defined set of journals reporting articles selected for validity and relevance); (4) online books (as they are not regularly updated); (5) original studies reported in medical journals, practice articles, abstracts of papers (ie, primary literature); (6) secondary literature (as it primarily comprises synthesized content, ie, level 2 of the “5S” levels of organization of evidence) [6]. No restrictions were placed on product development status, disease or medical area, or access or charging agreements.

**Table 1:** Definitions helpful to explain criteria for inclusions and exclusions

	Definition
Evidence-based practice	Evidence-based practice refers to the process of systematically finding, appraising, and using contemporaneous research findings as the basis for clinical decisions. Evidence-based practice follows four steps: formulate a clear clinical question from a patient's problem; search the literature for relevant clinical articles; evaluate (critically appraise) the evidence for its validity and usefulness; implement useful findings in clinical practice.
Point of care	Point of care refers to the specific point in the workflow when health professional and patient interact and applies to any service provided to patients at the bedside or during patients’ consultations.
Update	Update refers to renovation or integration of content within a maximum of five years.
Rapidly accessible	Rapidly accessible content should be easily available on searching by keywords or browsing by topics or alphabetically ordered menus. The research output should be sufficiently summarized and relevant.
Studies (primary literature)	Studies (primary literature) are publications that illustrate or comment on original scientific research findings, typically in journal articles.
Synthesis (secondary literature)	Syntheses (secondary literature) are published materials that provide an examination of recent or current literature. Review articles can cover a wide range of subject matter at various levels of completeness and comprehensiveness based on analyses of literature that may include research findings. The Cochrane Library is an example.
Synopsis	Synopsis is the selection and summary of clinically important articles in the medical literature (usually in specific fields), which include newly published, high-quality, clinically relevant original studies and systematic reviews. Online journal clubs are an example.
Summaries (tertiary literature)	Summaries (tertiary literature) are abstracts that integrate evidence from many sources (eg, primary literature, systematic reviews, and guidelines) to provide a full range of information on management options for a given health problem.
Systems (decision aid)	Systems (decision aids) are clinical information systems that integrate and summarize all relevant and important research evidence about a clinical problem and automatically link, through an electronic medical record, a specific patient's circumstances to the relevant information.
Literature surveillance alerting systems	Literature surveillance alerting systems provide regular monitoring of a defined set of journals and the reporting of article selection on the basis of validity and relevance (ie, Evidence UpDates, ACP Journal Club, InfoPOEMs)
Meta-lists	Meta-lists are information retrieval tools that contain links to other relevant sites on the Web. The links are usually collected by the meta-list site coordinator, who acts as a clearinghouse.
Search engine	Search engines are information retrieval tools aimed at searching for information on the whole Web or on medicine-specific websites. The strength of a medicine-specific search engine is its ability to filter out any sites that are not (according to programmed criteria) medical sites.
Guideline databases	Guideline databases are online repositories of clinical documents aimed at providing recommendations rather than information to clinicians (ie, SIGN, NICE).
Online books	Online books are electronic versions of paper-based publications. These are not regularly updated.

### Identification of EBP Point-of-Care Summary Providers

To our knowledge, there is no single repository of online information summaries. In order to retrieve relevant summary products we performed a Medline search using the following terms: (("evidence-based medicine"[Mesh]) AND ("information storage and retrieval"[Mesh])) AND (("online systems"[Mesh]) OR ("point-of-care systems"[Mesh])).

We collected additional information from the references cited in the papers retrieved. Google was extensively used as the search engine to explore products not reported in the medical literature but available on the market. The following terms were used: “medical information system,” “point of care,” and “evidence-based medicine.” We also screened several publisher and librarian association websites, such as the Council of Science Editors [11], the World Association of Medical Editors [12], the Medical Librarian Association [13], the European Association for Health Information and Libraries [14], and the American Medical Informatics Association [15]. Finally, we analyzed the publishing products presented at several scientific information conferences and exhibitions during the period 2006 through 2008, such as the London Online Information Expo and Medical Library Association Meeting and Exhibition.

We repeated our search and collection during the one-year period from January through December, 2008.

### Information Sought for Each EBP Point-of-Care Summary Provider

For each provider, two reviewers independently retrieved information through an analysis of the official website. As reported in detail below, for any EBP point-of-care summary provider we extracted general characteristics, volume and breadth of the conditions considered, and information regarding the quality of the editorial process and EBP approach to content development (evidence-based methodology). Decisions to select items describing these features were informed by evidence whenever possible. Detailed operational definitions are reported in Multimedia appendix 1.

The features selected were qualitatively described; for editorial and evidence-based methodology indicators, an empirical quantitative evaluation was also included in order to assign a score for each item and rank the EBP point-of-care summary providers. For each quality indicator a point score was assigned: 3 points if the quality indicator was completely fulfilled, 1 point if partially fulfilled or unclear, and 0 points if not fulfilled or not reported. (See Multimedia Appendices 2 and 3.) We arbitrarily decided to award 3 points instead of 2 for adequate fulfilment to give more weight to a more transparent and accountable reporting style and increase the variability within the sample. This policy was somewhat similar to the three-points-for-win rule in soccer [16].

#### General Characteristics

We first sought general information, such as product name, year of first release, and vendor and/or publisher. We also reported the marketing claim as stated in the homepage and/or in the “about us” section. We collected information on different formats (eg, online, desktop, or PDA) and whether the website is open-access or a subscription fee is required to access the whole content. In the latter case, we reported the costs for a single-user subscription per year and the types of subscription available, that is, single user, institutional, “à la carte” (ie, different products assembled in one subscription), or pay per view. We also described the primary target audience, for example, general practitioners, specialty physicians, or other health care workers who could benefit from the contents. (See Multimedia Appendix 1.)

#### Content Presentation

We described the content presentation in terms of type of output (ie, narrative or key point summaries or answers to clinical questions format), formal ontology of information, and output summary flexibility. We analysed whether the output included references, and if so, whether these were general, that is, suggestions of further sources on a particular topic, or specific, that is, they supported particular statements. We also explored whether, in addition to providing information, these summaries provided recommendations to practitioners, and if so, whether a formal grading system for the strength of the recommendations was used. Lastly, we sought whether the content of products included continuing medical education programmes or other educational resources and whether a plain language information document or handout had been specifically developed for patients. See Multimedia Appendix 1.

#### Breadth and Volume

We sought to describe the breadth of the medical conditions considered in terms of areas covered by the summaries (ie, general information, epidemiology, aetiology, physiopathology, diagnosis, treatment, follow up, and prognosis). As we were not able to identify a reliable measure of database volume (ie, comprehensiveness), we estimated the number of diseases covered by analyzing whether a random sample of chapters of the *International Classification of Diseases, Tenth Revision (ICD-10)* was represented in the product. This provided a rough proxy assessment of the comprehensiveness of each product (ie, its external validity). Of the 22 *ICD-10* chapters, 4 (20%) were randomly selected. These were: “Certain infectious and parasitic diseases,” “Diseases of the skin and subcutaneous tissue,” “Diseases of the genitourinary system,” and “External causes of morbidity and mortality.” We assessed whether sections (blocks) of diseases and conditions in these *ICD-10* chapters were covered in each EBP point-of-care summary [17].

In addition, we reported whether summary providers included information on topics other than medical conditions (eg, medical procedures and legal issues) and more complex technologies, such as electronic medical records, drug databases, and calculators.

#### Editorial Quality

To evaluate the methodological quality of the editorial process, we selected specific indicators of transparency: authorship, peer reviewing procedure, updating, disclosure of authors’ conflicts of interest, and commercial support of content development. To create an indicator of editorial quality, points were assigned: 3 points were assigned if the dimension was judged “adequate,” 1 point if judged “unclear,” and 0 points if judged “not adequate” or “not reported.” See Multimedia Appendix 2.

#### Evidence-based Methodology

To obtain information on the evidence-based approach to content development of each product, we specifically selected evidence-based methodology indicators. The indication of whether contents were based on a systematic literature search or surveillance aimed at identifying relevant, valid research evidence was considered of primary importance. The critical appraisal methodology was also judged, and we focused on the cumulative or discretionary approach to the evidence, reporting whether systematic reviews, particularly Cochrane reviews, were preferred over other types of publication. We also looked at the availability of a system to assess quality of evidence. Finally, if expert opinion was included in the content development, we analyzed whether this contribution could be easily recognized within the body of evidence. Similar to our method of scoring the quality of the editorial policy, we scored each indicator of the quality of the evidence-based approach: 3 points were assigned if the dimension was judged “adequate,” 1 point if judged “unclear,” and 0 points if judged “not adequate” or “not reported.” See Multimedia Appendix 3.

### Data Extraction

Data were extracted by two independent reviewers (authors RB and LT) who used an ad hoc predefined form. We obtained general features and information on the editorial policy and content development from a thorough analysis of the website pages that were freely available (ie, homepage, about us, editorial policy, and methodology description sections). When subscription to a product was not available at our institution, the free trial and sample topics were used to acquire further information on the content characteristics of the product and the type of output. We assumed that sample topics would likely provide users with the “best” of the product as these parts are often written with the most zeal and attention. When necessary, product editors were contacted by email. When we could not access the content, the products were excluded from the analysis. Disagreements were resolved by discussion between the reviewers and a referee (author LM).

We registered and stored within an electronic archive (December 2008) all the Web pages used to extract data.

### Analysis

Results are presented as median and interquartile ranges to describe the volume and quality indicator scores. The EBP point-of-care products were ranked on the basis of (1) the number of diseases covered (calculated as the number of diseases covered from those in a random sample of *ICD-10* chapters); (2) the editorial quality (defined on the basis of adherence to the items reported in Multimedia Appendix 2); and (3) the use of an evidence-based approach (defined on the basis of adherence to the items reported in Multimedia Appendix 3). The relationships between these factors were analyzed by applying the Spearman rank correlation coefficient.

## Results

**Figure 2:**
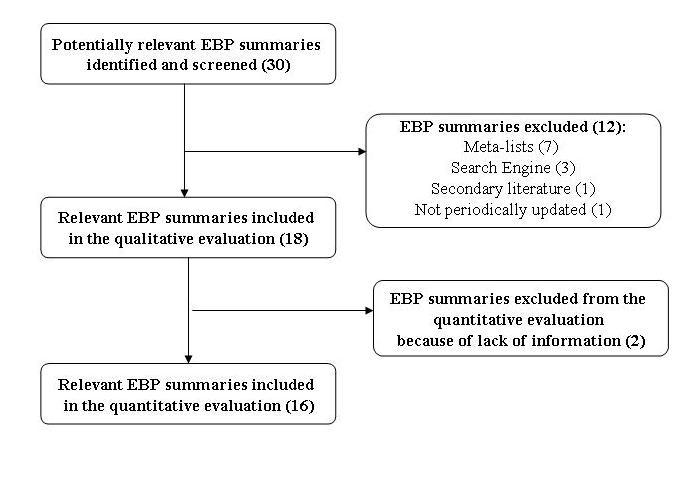
Flow diagram of the EBP point-of-care summary providers included in the analysis

From January to December 2008 we screened 30 eligible EBP point-of-care summary providers (Figure 2). Of these, 12 were excluded (for details see Multimedia Appendix 4), and 18 met our inclusion criteria and were qualitatively evaluated. Two summary providers (ZynxEvidence and Health Gate) were excluded from the quantitative analysis because of a lack of information on the website general pages and unavailability of sample chapters; we attempted to acquire the missing information from vendors but received no answer.

### Qualitative Analysis

General characteristics and summary content presentation features are summarized in Tables 2and 3. In the EBP point-of-care summaries breadth, we found no variability in the areas of medical conditions covered (data not tabulated). With the exception of Clinical Evidence, all the summary providers reported general information, epidemiology, aetiology, physiopathology, diagnosis, treatment, follow-up and prognosis for each topic but they differed in terms of widening and length. Clinical Evidence focused mainly on treatment alternatives; diagnosis and testing were not systematically covered. Several summary providers presented topics other than medical conditions. For example, 5-Minute Clinical Consult, ACP Pier, DynaMed, eMedicine, EBM Guidelines, First Consult included information about medical procedures; ACP Pier, GP Notebook included, ethical and legal issues; and Dynamed, Harrison’s Practice, Micromedex, and Pepid included drug information with summaries of product characteristics and pharmacokinetic interaction tables. Zynx Health presented complex content and integration with other technologies, such as electronic medical records while drug databases and calculators are distinctive of some other products, such as Micromedex and Pepid according to the shift from summary to systems described in the Haynes model [6].

**Table 2:** General characteristics of EBP point-of-care summary providers

Product Name (URL)	Year of Release	Vendor/ Publisher	Marketing Claim	Fee-based/ Open Access	Type of Subscription	Format	Annual Cost for Single User Account for year	Target Users
5-Minute Clinical Consult (www.5minuteconsult.com)	Not reported	Wolters Kluwer, Lippiccott Williams and Wilkins	Updated regularly for quick reference at the point of care.	Fee-based	Single user	Online, PDA, smartphone, print	US $89.90	Not reported
ACP Pier (www.acponline.org)	Not reported	American College of Physicians	Find authoritative, evidence-based guidance to improve clinical care	Open access to ACP members	Not Applicable	Online and PDA	Not Applicable	Internal medicine specialists
BestBETs (www.bestbets.org/)	1996	Department of Emergency Medicine, Manchester Royal Infirmary	… provide rapid evidence-based answers to real-life clinical questions, using a systematic approach to reviewing the literature.	Open access	Not Applicable	Online and print	Not Applicable	Emergency medicine specialists
CKS (www.cks.nhs.uk)	1998	NHS	Safe practical clinical answers- fast	Open access	Not applicable	Online and print	Not applicable	GPs, nurses, pharmacists, students; medical librarians
Clinical Evidence (www.clinicalevidence.bmj.com)	1999	BMJ Publishing group	The international source of the best available evidence on the effects of common clinical interventions	Fee-based	Single user, institutional, pay per view, season ticket	online, print (handbook), PDA	£137/€203/ US $260	GPs, specialists
DynaMed (www.ebscohost.com/dynamed/)	Not reported	EBSCO Publishing	Designed for use at the point-of-care, providing best available evidence and updated daily.	Fee-based	Single user, institutional	Online, PDA	US $350	GPs, specialists
eMedicine (www.emedicine.medscape.com/)	1996	WebMD- Medscape	Continually updated clinical reference…the most authoritative and accessible point-of-care medical reference available to physicians.	Open access	Not applicable	Online	Not applicable	GPs and other health care professionals.
eTG complete (www.tg.org.au)	1978	Therapeutic Guidelines Limited (Australia)	Therapeutic Guidelines…evidence in context	Fee-based	Single user, student subscription	Online, desktop, print, PDA	A$300	Not reported
EBM Guidelines (http://ebmg.wiley.com)	1989	Wiley Blackwell Interscience and Duodecim	Easy to use clinical guidelines supported by sound scientific evidence	Fee-based	Single user, institutional	Online, print, PDA	US $255	GPs
First Consult (www.mdconsult.com)	1997	Elsevier	Evidence-based answers for the point of care	Fee-based	Single user, institutional	Online, PDA	US $449 "Core + first consult"	GPs, specialists
GP Notebook (www.gpnotebook.co.uk)	1995	Oxbridge Solutions Ltd	A UK medical reference on the world wide web	Open Access	Not applicable	Online	Not applicable	GPs

Harrison’s Practice (www.harrisonspractice.com)	Not reported	Mc Graw Hill	Answers on demand at the point of care	Fee-based	Single user, institutional	online, PDA, wireless version	US $325	GPs, Internal Medicine Specialists
Health Gate (www.healthgate.com)	Not reported	HealthGate Data Corporation	The latest evidence-based clinical information	Fee-based	Not reported	Online	Not reported	Providers, payers, employers, and patients
Map of Medicine (www.mapofmedicine.com)	2001	Hearst Corporation	Support for clinical practice national, local and personal evidence-based content broad in scope	Open access to all NHS staff in England and Wales.	Not applicable	Online	Not applicable	GPs and other health professionals
Micromedex (www.micromedex.com)	Not reported	Thomson Reuters	Evidence-based answers to support your disease management and treatment decisions.	Fee-based	Not reported	Online, PDA	Contact for pricing	GPs, specialists, other health professionals. Medical school faculty and students, librarians
Pepid (www.pepid.com)	1994	Pepid LLC	The only “all-in-one” point-of-care medical reference tool available on the Internet	Fee-based	Single user, institutional	Online, PDA, Mobile Wireless	US $199.95 Primary Care Plus PCP	GPs, specialists
UpToDate (www.uptodate.com)	1992	UpToDate, Inc	UpToDate is an evidence-based, peer-reviewed information resource	Fee-based	single user institutional, patient subscription	Online, desktop, PDA	US $495	GPs, specialists
ZynxEvidence (www.zynxhealth.com/)	Not reported	Zynx Health Incorporated	Evidence-based health care. Informed decision. Improved care.	Fee-based	Not reported	Not reported	Not reported	GPs, specialists

**Table 3:** Content presentation of EBP point-of-care summary providers

	Output Presentation						Education		
Name	Type of Output	Formal Ontology	Summary Flexibility	References	Intent to Recommend	Strength of Recommendation Formal System	CME Programs	Other Educational Material	Patient Handout
5-Minute Clinical Consult	Key point summary	Yes	No	Yes, general	Yes	No	No	No	Yes
ACP Pier	Key point summary	Yes	Yes	Yes, specific	Yes	No	Yes	No	Yes
BestBETs	Answers to clinical questions	Yes	No	Yes, specific	No	No	No	Yes, methodology	No
CKS	Key point summary	Yes	Yes	Yes, general	Yes	No	No	No	Yes
Clinical Evidence	Narrative summaries on clinical questions	Yes	Yes	Yes, specific	No	No	Yes	Yes, statistics and methodology	Yes
DynaMed	Key point summary	Yes	No	Yes, specific	Yes	Yes	Yes	No	No
eMedicine	Book chapter-like summary	Yes	No	Yes, general	Yes	No	Yes	No	No
eTG complete	Book chapter- like summary	No	No	Yes, general	Yes	No	No	No	No
EBM Guidelines	Key point summary	Yes	No	Yes, specific	Yes	No	No	No	No
First Consult	Key point summaries	Yes	Yes	Yes, general	Yes	No	Yes	No	Yes
GP Notebook	Book chapter- like and key point summaries	No	No	Yes, general	No	No	Yes	No	No
Harrison’s Practice	Key point summaries	Yes	No	Yes, general	Yes	No	Yes	No	No
Health Gate	No information	No information	No information	Yes, general	No information	No	No	No	Yes
Map Of Medicine	Clinical pathways	Yes	Yes	Yes, specific	Yes	No	No	No	No
Micromedex	Key point summaries	Yes	Yes	Yes, specific	Yes	Yes	No	No	No
Pepid	Key point summaries	Yes	No	No	Yes	No	No	Yes	No
UpToDate	Book chapter- like summaries	Yes	No	Yes, specific	Yes	Yes	Yes	No	Yes
ZynxEvidence	Key point summary	Yes	No	No information	Yes	No information	No	No	No

#### Quantitative Analysis

**Figure 3:**
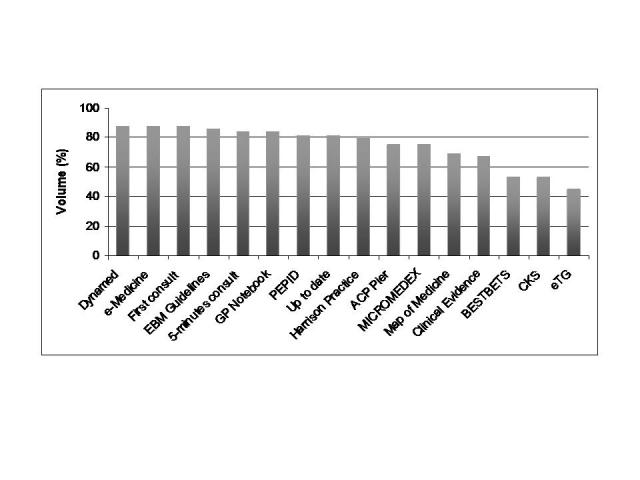
EBP point-of-care summary provider volume estimated on four random chapters of the ICD-10 classification

The EBP point-of-care summary volume based on four random samples of *ICD-10* chapter analysis is estimated in Figure 3. The median coverage volume was 80.6% (interquartile range: 68.9-84.2%). There were large differences among summaries, with DynaMed, eMedicine, and First Consult being the most comprehensive (88%) and eTG complete the least (45%).

Editorial policy quality and evidence-based methodology are summarized in Tables 4 and 5. The median scores were 8.0 (interquartile range 5.8-10.3) and 10.0 (interquartile range 1.0-12.8) on a 15-point scale.

EBP point-of-care summary provider scores were ranked according to volume, editorial, and EB methodology scores (see Multimedia Appendix 5). Table 4 shows the scores for each provider for editorial quality; Table 5 shows the scores for EBP methodology; and Figure 3 displays the results of the analysis of volume (ie, comprehensiveness).

**Figure 4:**
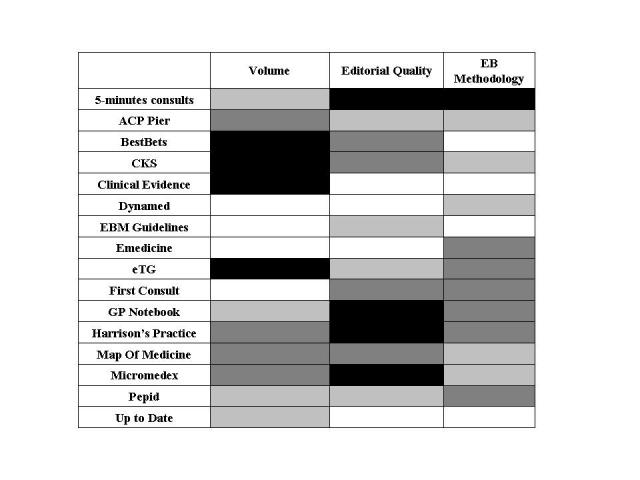
EBP point-of-care summary provider ranking for volume, editorial quality and evidence-based methodology. (Black represents the bottom quartile; dark grey represents the low intermediate quartile; light grey represent the high intermediate quartile; and white represents the top quartile.)

Displayed together in Figure 4 are the EBP point-of-care summary provider rankings for volume, editorial quality and EB methodology. As is shown, DynaMed, EBM Guidelines, and UpToDate scored in the top quartile for two out of three variables and in the second quartile for the third of these variables. However, no association was found between the pairs of variables for each EBP point-of-care summary provider (Spearman rank correlations: editorial quality and volume, *r* = -0.001, *P* = .99; EB methodology and volume *r*  = -0.19, *P* = .48; editorial and EB methodology *r* = 0.43, *P* = .09).

A brief presentation of these results is reported in Multimedia Appendix 6.

**Table 4:** Editorial quality of EBP point-of-care summary providers

Name	Authorship (Points)	Reviewing (Points)	Updating (Points)	Authors’ Conflict of Interest (Points)	Commercial Support for Content Development (Points)	Editorial Quality Score
Clinical Evidence	Yes (3)	Yes (3)	Yes (3)	Yes, implemented and reported (3)	Not accepted (3)	15
UpToDate	Yes (3)	Yes (3)	Yes (3)	Yes, implemented and reported (3)	Not accepted (3)	15
eMedicine	Yes (3)	Yes (3)	Yes (3)	Yes, implemented and reported (3)	Accepted and disclosed (1)	13
DynaMed	Unclear (1)	Unclear (1)	Yes (3)	Yes, implemented and reported (3)	Not accepted (3)	11
eTG complete	Unclear (1)	Yes (3)	No (0)	Yes, implemented and reported (3)	Not accepted (3)	10
ACP Pier	Yes (3)	Yes (3)	Yes (3)	No information (0)	No information (0)	9
EBM Guidelines	Yes (3)	Yes (3)	Yes (3)	No information (0)	No information (0)	9
Pepid	Yes (3)	Yes (3)	Yes (3)	No information (0)	No information (0)	9
First Consult	Yes (3)	Unclear (1)	Yes (3)	No information (0)	No information (0)	7
BestBETs	Yes (3)	Yes (3)	No (0)	No information (0)	No information (0)	6
CKS	No (0)	Yes (3)	Yes (3)	No information (0)	No information (0)	6
Map Of Medicine	No (0)	Yes (3)	Yes (3)	No information (0)	No information (0)	6
Micromedex	No (0)	Yes (3)	Unclear (1)	Yes, implemented but not reported (1)	No information (0)	5
5-Minute Clinical Consult	Yes (3)	No (0)	Unclear (1)	No information (0)	No information (0)	4
GP Notebook	No (0)	Unclear (1)	Yes (3)	No information (0)	No information (0)	4
Harrison’s Practice	No (0)	No (0)	Yes (3)	No information (0)	No information (0)	3

**Table 5:** Evidence-based methodology of EBP point-of-care summary providers

Name	Literature Search/ Literature Surveillance (Points)	Cumulative vs Discretionary Approach (Points)	Critical Appraisal (Points)	Formal Grading of Evidence (Points)	Cite Expert Opinion (Points)	Evidence-Based Methodology Score
BestBETs	Yes (3)	Yes (3)	Yes (3)	Yes (3)	Yes (3)	15
Clinical Evidence	Yes (3)	Yes (3)	Yes (3)	Yes (3)	Yes (3)	15
EBM Guidelines	Yes (3)	Yes (3)	Yes (3)	Yes (3)	Yes (3)	15
UpToDate	Yes (3)	Yes (3)	Yes (3)	Yes (3)	Yes (3)	15
DynaMed	Yes (3)	No (0)	Yes (3)	Yes (3)	Yes (3)	12
Map Of Medicine	Yes (3)	Yes (3)	Yes (3)	No (0)	Yes (3)	12
Micromedex	Yes (3)	Yes (3)	Unclear (1)	Yes (3)	Unclear (1)	11
ACP Pier	Yes (3)	No (0)	Yes (3)	Yes (3)	Unclear (1)	10
CKS	Yes (3)	Yes (3)	Unclear (1)	No (0)	Yes (3)	10
Pepid	Unclear (1)	Unclear (1)	No (0)	No (0)	No (0)	2
eMedicine	Unclear (1)	No (0)	No (0)	No (0)	No (0)	1
eTG complete	Unclear (1)	No (0)	No (0)	No (0)	No (0)	1
First Consult	Unclear (1)	No (0)	No (0)	No (0)	No (0)	1
GP Notebook	Unclear (1)	No (0)	No (0)	No (0)	No (0)	1
Harrison’s Practice	Unclear (1)	No (0)	No (0)	No (0)	No (0)	1
5-Minute Clinical Consult	No (0)	No (0)	No (0)	No (0)	No (0)	0

## Discussion

### Summary of Key Findings

As of December 2008, we had found 18 EBP point-of-care summary providers. This suggests that several publishing groups and public health organizations are interested in investing time and resources into the development of these products. The overall characteristics of these products tended to vary, and evaluation of their quality is still in its infancy despite the emerging consensus that such information tools are professionally and scientifically essential [4]. Only a few products satisfied our criteria, with none excelling in all. Thus, at present, no clear set of dimensions for deciding among different products can be drawn. The choice of an information tool will depend on the properties of the resource and users’ preferences according to the personal weight attached to different rankings.

### Our Study in Context

One mainstay of evidence-based information mastery is the combination of tools that filter literature for relevance and validity and present summaries easily and in a quickly accessible form at the point of care [3]. Since doctors have significant information needs in their practice [18,19], an important question is whether all these products are reliable and really improve access to high-quality information. While many user-centred or experience/satisfaction analyses have been published [20-25], our evaluation aimed at providing an explicit way to assess the available products other than relying on potentially misleading marketing claims by vendors.

We developed a content validity scale using an evidence-based approach whenever possible. Desirable dimensions were included if there was evidence that not addressing that particular dimension would result in an increased risk of bias. Dimensions were also included where it was clear that information about that dimension was necessary to appraise the reliability of a point-of-care product. For some quality indicators, such as the literature retrieval process and updating, we borrowed our criteria from research on systematic review reporting methods [26,27], assuming that these also would apply to the further synthesized information tools that we included in our review. Other scale dimensions, such as authors’ conflicts of interest and peer review, were based on peer-reviewed medical journals’ policies, extensively debated during the past years [28-30]. Other dimensions that we measured, such as intent to recommend, were included because we judged them to be important, but these were not clearly based on prior research. Only 20% of the products that included recommendations formally graded the strength of the recommendations, whereas doing so is essential to assure transparency and reliability of recommendations [31].

### Limitations of This Study

One of the limitations of our study stems from a lack of a clear definition of these products which could have led to a possible selection bias. We set eligibility criteria to select evidence-based summary products defined as portable and comprehensive [1] that Haynes et al would categorize as a summary [6]. Moreover, summary provider eligibility was independently evaluated by two authors. Our study is only a first attempt toward a more comprehensive assessment of this rapidly evolving field. The number of EBP point-of-care summary providers is increasing; in the first months of 2009 at least three vendors, the Journal of the American Medical Association (http://jamaevidence.com), the British Medical Journal (www.bestpractice.bmj.com) and the UK National Health Service (www.evidence.nhs.uk/) launched new point-of-care products. These new products were not included in our survey because they were launched outside the considered time frame.

The major limitation of our study was the arbitrariness of the scoring system. We chose a continuous scale instead of a classical star rating system to allow scores on individual categories to be correlated. Category scores have not been added to make an overall score. Scores allow readers to group EBP point-of-care summary providers according to quality and to detect top performers within categories. Our scoring system can be considered a preliminary approach to rating EBP point-of-care summary providers; other categories could be added.

We did not formally analyze website navigability and usability as this was beyond the scope of our study. Such an analysis might be valuable from the users’ perspective because information on the Web can be communicated in many ways—such as diagrams, animations, and linked pages—which may improve comprehension. These analyses should be carefully interpreted as they suffer from the multiplicity bias as occurs when users are asked to compare known systems with new ones. EBP point-of-care summaries also largely differ according to the comprehensiveness of each topic. Choosing a random sample of *ICD-10* chapters as a proxy of the comprehensiveness of a summary may not necessarily be representative of what each provider offers. However, comprehensiveness is a crucial aspect of any information tool when used to answer clinical questions. Further research on the comprehensiveness of these information tools is needed.

### Relationships Between Volume, Editorial Quality, Evidence-based Methodology

None of the associations we postulated turned out to be statistically significant. Thus, on the basis of the criteria we used, editorial quality, evidence-based methodology, and volume appear to be independent. For example, BestBETs scored among the worst on volume (comprehensiveness), with an intermediate score for editorial quality, and the highest score for evidence-based methodology. The search for associations between various desirable factors can be seen as “work in progress,” suggesting that publishers have to balance these aspects, and achieving excellence in all three aspects is difficult.

### Implications for Editorial/Publishing Groups

In the global trend for point-of-care products to inform clinical practice, there is room for improving the quality and increasing the coverage of diseases. Publishers should provide users (or purchasers in general) with transparent, easily accessible, and rigorously determined information regarding editorial processes and content development. Our assessment is intertwined with the quality of reporting. It is possible that publishers favored conciseness of information on their websites and omitted important editorial and methodological details. For instance a publisher may plan to disclose author conflicts of interest, but then does not report this key information on its website, thus diminishing the trustworthiness of its product.

Efforts have been made in the last two decades to improve the quality of reporting of the results of randomized controlled trials and systematic reviews [26,27,32]. However, there is still evidence that methods and reporting can be improved [33-35]. The experience obtained in the field of primary research can be applied to EBP point-of-care information summaries, considering that these point-of-care products are still in the early development. Important initiatives to improve the reporting of health care research, such as the EQUATOR Network [36,37] should also include initiatives to improve point-of-care products.

### Implications for Clinicians

At present, clinicians who want to select an EBP point-of-care summary to use regularly need to find a balance among several desirable characteristics to inform their choice: according to our criteria, no product appears to be the best. Faced with a choice of summaries, one criterion should prevail. The judgement is complex because in addition to various desirable criteria, many other dimensions could be attractive and drive the choice, such as whether the summary can be used for continuing medical education, contains information addressed to patients, or can be integrated with more sophisticated technologies. Having access to high-quality and well-summarized evidence-based information will not answer all the questions that arise in the doctor-patient relationship, but these summaries help doctors to identify the best options in therapy, diagnosis, or prognosis for their patients. Even the most innovative information system must rely on sound evidence to improve clinical practice; the technology is only the vehicle to make the information accessible. Quality indicators that can be used to evaluate new EBP point-of-care summary providers can be valuable for clinicians, but these can also be useful for librarians, hospital managers, and policy makers who must choose the most appropriate point-of-care summaries to meet their needs.

## Multimedia Appendix 1

Operational definitions adopted for this study

## Multimedia Appendix 2

Instrument to measure editorial policy quality (max 15 points)

## Multimedia Appendix 3

Instrument to measure editorial policy quality (max 15 points)

## Multimedia Appendix 4

Online EBP information resources excluded and reasons

## Multimedia Appendix 5

EBP point-of-care summary provider scores and ranks according to volume, editorial quality, and evidence-based methodology

## Multimedia Appendix 6

A review of online evidence-based practice point-of-care information summary providers

## Abbreviations

EBP: evidence-based practice
ICD-10: International Classification of Diseases, Tenth Revision
