# Supplementary material for: A Review of Online Evidence-based Practice Point-of-Care Information Summary Providers
Source: J Med Internet Res. 2010 Jul 7;12(3):e26. doi: 10.2196/jmir.1288 (PMC2956323; doi:10.2196/jmir.1288)
Supplement: Supplementary file 1 [file jmir_v12i3e26_app1.pdf]

Multimedia Appendix 1. Operational definitions adopted for this study

General characteristics of EBP point-of-care summary providers (Table 2)

- Name
- Year of release
- Vendor/Publisher: institutions, editors, company providing and publishing resources.
- (Marketing) claim: as stated directly in the website homepage or “About us” section.
- Fee based/Open access: if a paid subscription fee is required to access the whole content of the resource.
- Type of subscription: single user, institutional, “à la carte”, pay per view, etc.
- Format: description of the different product formats (i.e. online, desktop, PDA, etc.)
- Annual cost: for a single-user subscription per year.
- Target: to whom the information tool is mainly addressed (general practitioners, specialty physicians, etc.). We also reported if it is stated that other health care professionals can benefit from that information tool contents.

Content presentation of EBP point-of-care summary providers (Table 3)

- Output presentation
    - Type of output: book chapter-like summaries, key point summaries, answers to clinical questions, other.
    - Formal ontology of information: extent to which the tool is optimised to provide consistent and schematic information (through domains – e.g. drugs, and classes – e.g. antibiotic) that can be easily accessed during a consultation. Other examples of domains and classes are: benefit/overall survival; harm/ neurotoxicity; complementary medicine / acupuncture.[1-3] (yes/no)
    - Summary flexibility: ability to retrieve brief relevant information and in-depth content by opening or expanding a single section or category. (yes/no)
    - References/link to bibliography: if general references suggested to deepen particular topics, or references supporting any reported statements are included. (Yes specific/Yes, general/No)

- Intent to recommend: extent to which the summary gives clinical guidance to direct action as well as providing research results (from facts to acts). (yes/no)

- Strength of recommendation formal grading: the use of a formal system to grade the strength of recommendations.[4] (yes/no)
- Education programme
  - Continuing medical education (CME) programmes: link to CME systems with the possibility of collecting CME credits. (yes/no)
  - Additional education materials: e.g. statistical and methodological supporting material. (yes/no)
- Patients handout: a plain language content specifically developed for patients and hosted by the website (outer links were excluded). (yes/no)

Editorial quality (Table 4)

- Authorship: clear indication of the author(s) of a specific content reported in the output. A generic "editorial team" was considered unclear.
- Reviewing process: a detailed description of the procedures aimed at assessing and ensuring the scientific quality of output (review process by external peer reviewers and/or by editors).[5]
- Updating: frequency of content updating (continuously, periodically, once a year, etc). Content updated within two years was considered adequate as a sign for updating occurred within two years for 23% of reviews.[6]
- Authors' conflict of interests: whether a formal policy on authors' commercial conflict of interests is implemented and this information is reported. [7, 8]
- Commercial support: to what extent commercial support and advertising are accepted in the content development policy. [8, 9]

Evidence-based Methodology (Table 5)

- Literature search/surveillance: indication of whether contents are written on the basis of a specific systematic literature search based on explicit search strategies and aimed at identifying relevant and valid articles or if systematic tracking of the relevant and valid articles based on predefined sample of leading journal and journal review services is utilised.[10]
- Cumulative vs. discretionary approach: whether content is preferably written on the basis of systematic reviews, particularly Cochrane Reviews rather than other publications.[11]

- Critical appraisal methodology: the use of standard and transparent methods to assess articles' validity.[12]
- Grading of evidence quality: if a formal system is implemented to grade the level of evidence.[4]
- Cite expert opinions: if statements based on experts' opinions are easily recognisable compared to study data and results.[13]

References

1. de Bruijn B, Martin J. Getting to the (c)ore of knowledge: mining biomedical literature. Int J Med Inform 2002 Dec 4;67(1-3):7-18.
2. Guarino N. Formal Ontology in Information SystemsProceedings of FOIS’98, Trento, Italy, 6-8 June 1998 Amsterdam, IOS Press, pp 3-15, 1998.
3. Shahar Y, Young O, Shalom E, Galperin M, Mayaffit A, Moskovitch R, Hessing A. A framework for a distributed, hybrid, multiple-ontology clinical-guideline library, and automated guideline-support tools. J Biomed Inform 2004 Oct;37(5):325-44.
4. Atkins D, Eccles M, Flottorp S, Guyatt GH, Henry D, Hill S, Liberati A, O'Connell D, Oxman AD, Phillips B, Schunemann H, Edejer TT, Vist GE, Williams JW, Jr. Systems for grading the quality of evidence and the strength of recommendations I: critical appraisal of existing approaches The GRADE Working Group. BMC Health Serv Res 2004 Dec 22;4(1):38.
5. Jefferson T, Rudin M, Brodney Folse S, Davidoff F. Editorial peer review for improving the quality of reports of biomedical studies. Cochrane Database Syst Rev 2007(2):MR000016.
6. Shojania KG, Sampson M, Ansari MT, Ji J, Doucette S, Moher D. How quickly do systematic reviews go out of date? A survival analysis. Ann Intern Med 2007 Aug 21;147(4):224-33.
7. Boyd EA, Bero LA. Improving the use of research evidence in guideline development: 4. Managing conflicts of interests. Health Res Policy Syst 2006;4:16.
8. Krimsky S, Rothenberg LS. Financial interest and its disclosure in scientific publications. JAMA 1998 Jul 15;280(3):225-6.
9. Smith R. Medical journals are an extension of the marketing arm of pharmaceutical companies. PLoS Med 2005 May;2(5):e138.
10. Lefebvre C, Manheimer E, Glanville J. Chapter 6: Searching for Studies
In: Higgins JPT, Green S (editors) Cochrane Handbook for Systematic Reviews of Interventions Wiley, 2008
11. McKinlay RJ, Cotoi C, Wilczynski NL, Haynes RB. Systematic reviews and original articles differ in relevance, novelty, and use in an evidence-based service for physicians: PLUS project. J Clin Epidemiol 2008 May;61(5):449-54.
12. Higgins J, Altman D, (editors). Chapter 8: Assessing risk of bias in included studies: In: Higgins JPT, Green S (editors) Cochrane Handbook for Systematic Reviews of Interventions Wiley, 2008
13. Antman EM, Lau J, Kupelnick B, Mosteller F, Chalmers TC. A comparison of results of meta-analyses of randomized control trials and recommendations of clinical experts. Treatments for myocardial infarction. JAMA 1992 Jul 8;268(2):240-8.
