# Supplementary material for: A Review of Online Evidence-based Practice Point-of-Care Information Summary Providers
Source: J Med Internet Res. 2010 Jul 7;12(3):e26. doi: 10.2196/jmir.1288 (PMC2956323; doi:10.2196/jmir.1288)
Supplement: Supplementary file 2 [file jmir_v12i3e26_app2.pdf]

Multimedia Appendix 2. Instrument to measure editorial policy quality (max 15 points)

1. Is/Are the content author(s) clearly stated**?**

   Score: 3 for "clearly stated", 1 for "unclear", and 0 for "not stated"
2. Has peer reviewing been done?

   Score: 3 for "done", 1 for "unclear", 0 for "not done"
3. Is content updating adequate (within two years)?

   Score: 3 for "yes", 1 for "unclear", 0 for "no"
4. Is a formal policy implemented and reported on authors' commercial conflict of interests?

   Score: 3 for "yes, implemented and reported", 1 for "implemented but not reported", 0 for "conflict of interests not requested (no information)"
5. Does the website accept any type of commercial support?

   Score: 3 for "not accepted", 1 for "accepted but disclosed", 0 for "no information"
