# Supplementary material for: A Review of Online Evidence-based Practice Point-of-Care Information Summary Providers
Source: J Med Internet Res. 2010 Jul 7;12(3):e26. doi: 10.2196/jmir.1288 (PMC2956323; doi:10.2196/jmir.1288)
Supplement: Supplementary file 3 [file jmir_v12i3e26_app3.pdf]

Multimedia Appendix 3. Instrument to measure EB methodology (max 15 points)

1. Is a systematic literature search or surveillance the basis of content development?

   Score: 3 for “yes”, 1 for “unclear”, and 0 for “no”
2. Is the critical appraisal method fully described?

   Score: 3 for “yes”, 1 for “unclear”, 0 for “no”
3. Are systematic reviews preferred over other types of publication?

   Score: 3 for “yes”, 1 for “unclear”, 0 for “no”
4. Is there a system for grading the quality of evidence?

   Score: 3 for “yes”, 1 for “unclear”, 0 for “no”
5. When expert opinion is included is it easily recognisable over studies’ data and results ?

   Score: 3 for “yes”, 1 for “unclear”, 0 for “no”
