# Supplementary material for: A Review of Online Evidence-based Practice Point-of-Care Information Summary Providers
Source: J Med Internet Res. 2010 Jul 7;12(3):e26. doi: 10.2196/jmir.1288 (PMC2956323; doi:10.2196/jmir.1288)
Supplement: Supplementary file 4 [file jmir_v12i3e26_app4.pdf]

Multimedia Appendix 4. Online EBP information resources excluded and reasons

EBP point-of-care summary	Reason for exclusion
ATTRACT	Not periodically updated
TRIP	Search engine
STAT! Ref	Meta-list
Evidence-Based Medicine Reviews (EBMR)	Meta-list
Essential Evidence Plus	Meta-list
EBM Search engine	Search engine
The Cochrane Library	Secondary literature
Clinical Information Access Program (CIAP)	Meta-list
CLIN-eGUIDE	Meta-list
Evidence Matters	Search engine
MedLine Plus	Meta-list
AccessMedicine	Meta-list
