# Supplementary material for: A Review of Online Evidence-based Practice Point-of-Care Information Summary Providers
Source: J Med Internet Res. 2010 Jul 7;12(3):e26. doi: 10.2196/jmir.1288 (PMC2956323; doi:10.2196/jmir.1288)
Supplement: Supplementary file 5 [file jmir_v12i3e26_app5.pdf]

**Multimedia Appendix 5.** EBP point-of-care summary scores and ranks according to volume, editorial quality, and evidence-based methodology

| Name | Volume | | Editorial quality | | EB methodology | |
|---|---|---|---|---|---|---|
| | % | Rank | Score | Rank | Score | Rank |
| **5-minutes consults** | 83.7 | 5.5 | 4 | 14.5 | 0 | 16 |
| **ACP Pier** | 75.5 | 10.5 | 9 | 7 | 10 | 8.5 |
| **BestBets** | 53.1 | 14.5 | 6 | 11 | 15 | 2.5 |
| **CKS** | 53.1 | 14.5 | 6 | 11 | 10 | 8.5 |
| **Clinical Evidence** | 67.3 | 13 | 15 | 1.5 | 15 | 2.5 |
| **Dynamed** | 87.8 | 2 | 11 | 4 | 12 | 5.5 |
| **EBM Guidelines** | 85.7 | 4 | 9 | 7 | 15 | 2.5 |
| **Emedicine** | 87.8 | 2 | 13 | 3 | 1 | 13 |
| **eTG** | 44.9 | 16 | 10 | 5 | 1 | 13 |
| **First Consult** | 87.8 | 2 | 7 | 9 | 1 | 13 |
| **GP Notebook** | 83.7 | 5.5 | 4 | 14.5 | 1 | 13 |
| **Harrison's Practice** | 79.6 | 9 | 3 | 16 | 1 | 13 |
| **Map Of Medicine** | 69.4 | 12 | 6 | 11 | 12 | 5.5 |
| **Micromedex** | 75.5 | 10.5 | 5 | 13 | 11 | 7 |
| **Pepid** | 81.6 | 7.5 | 9 | 7 | 2 | 10 |
| **Up to Date** | 81.6 | 7.5 | 15 | 1.5 | 15 | 2.5 |
