# Supplementary material for: A Review of Online Evidence-based Practice Point-of-Care Information Summary Providers
Source: J Med Internet Res. 2010 Jul 7;12(3):e26. doi: 10.2196/jmir.1288 (PMC2956323; doi:10.2196/jmir.1288)
Supplement: Supplementary file 6 [file jmir_v12i3e26_app6.ppt]

## Slide 1
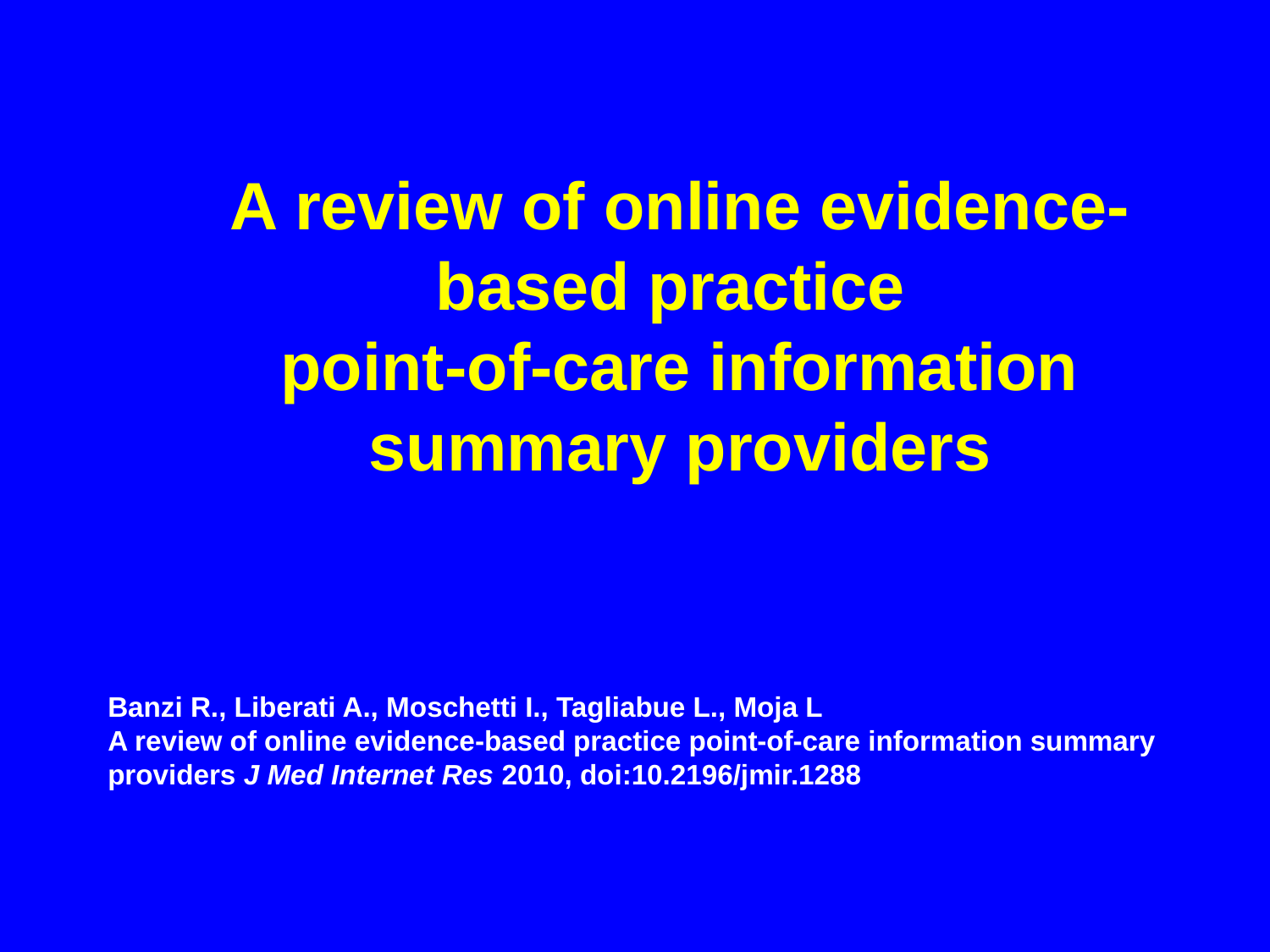

A review of online evidence-based practice
point-of-care information summary providers
# Banzi R., Liberati A., Moschetti I., Tagliabue L., Moja L A review of online evidence-based practice point-of-care information summary providers J Med Internet Res 2010, doi:10.2196/jmir.1288

## Slide 2
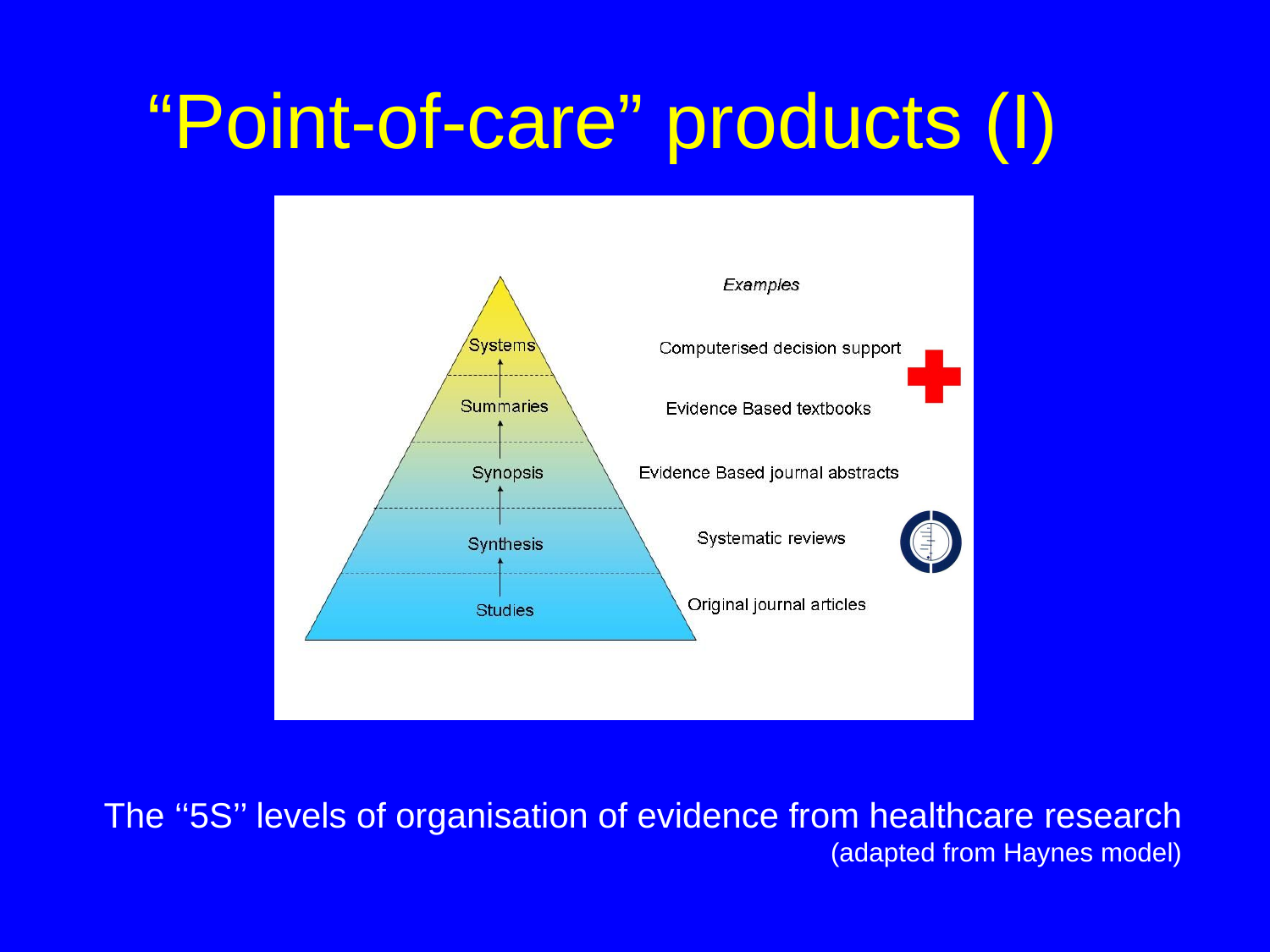

# “Point-of-care” products (I)
The ‘‘5S’’ levels of organisation of evidence from healthcare research (adapted from Haynes model)

## Slide 3
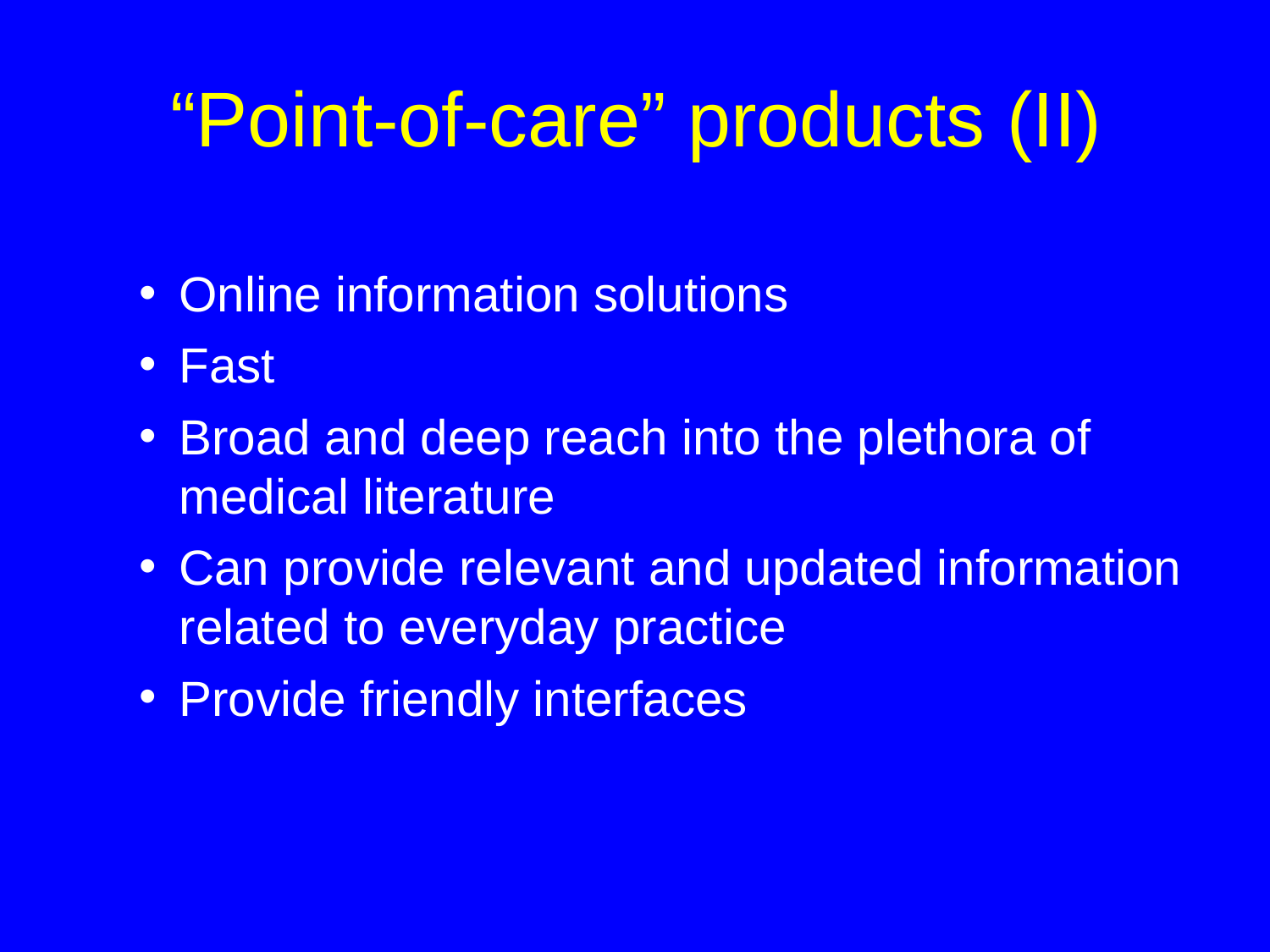

# “Point-of-care” products (II)
Online information solutions
Fast
Broad and deep reach into the plethora of medical literature
Can provide relevant and updated information related to everyday practice
Provide friendly interfaces

## Slide 4
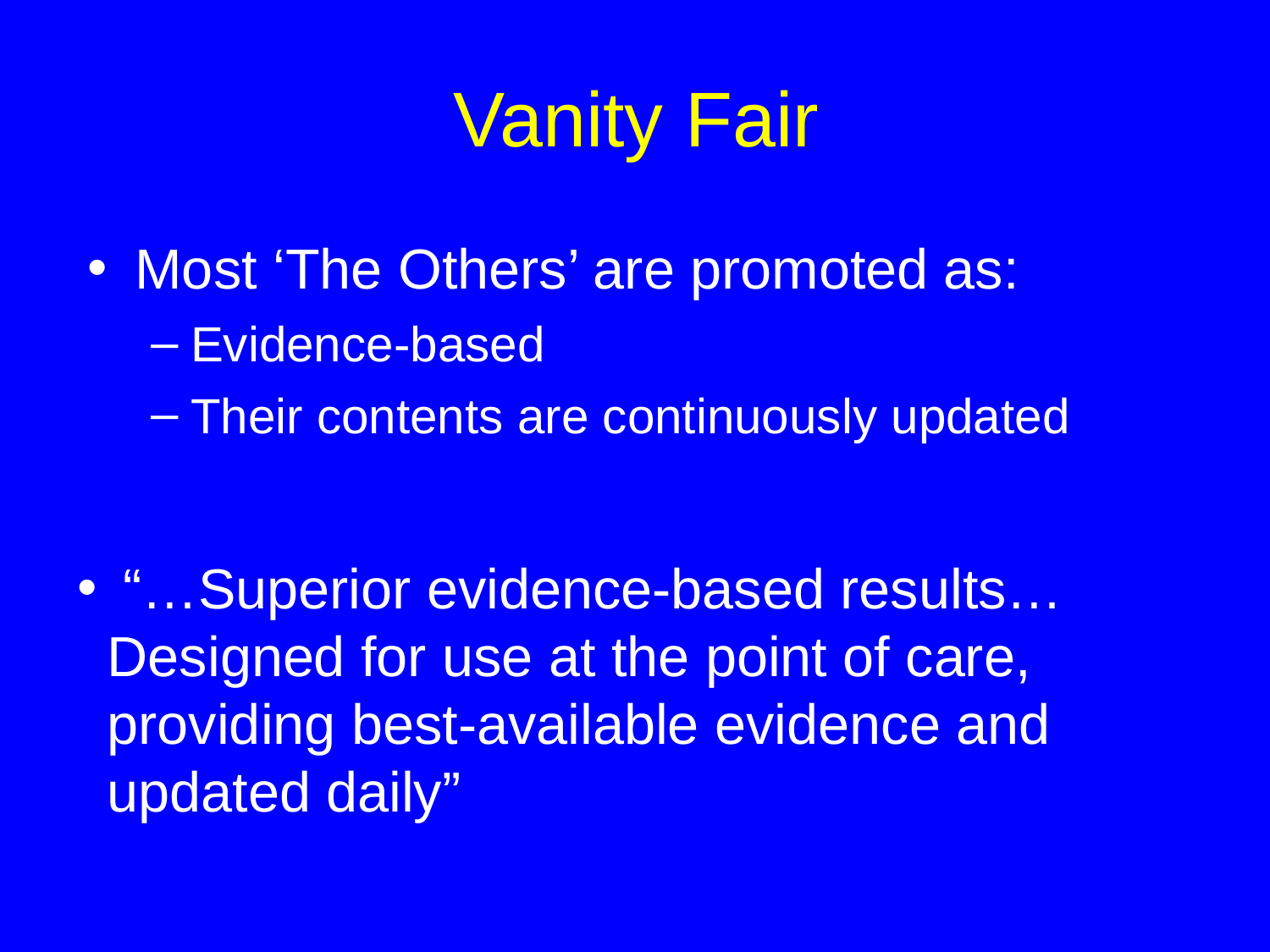

Vanity Fair
# Most ‘The Others’ are promoted as:
Evidence-based
Their contents are continuously updated
 “…Superior evidence-based results… Designed for use at the point of care, providing best-available evidence and updated daily”

## Slide 5
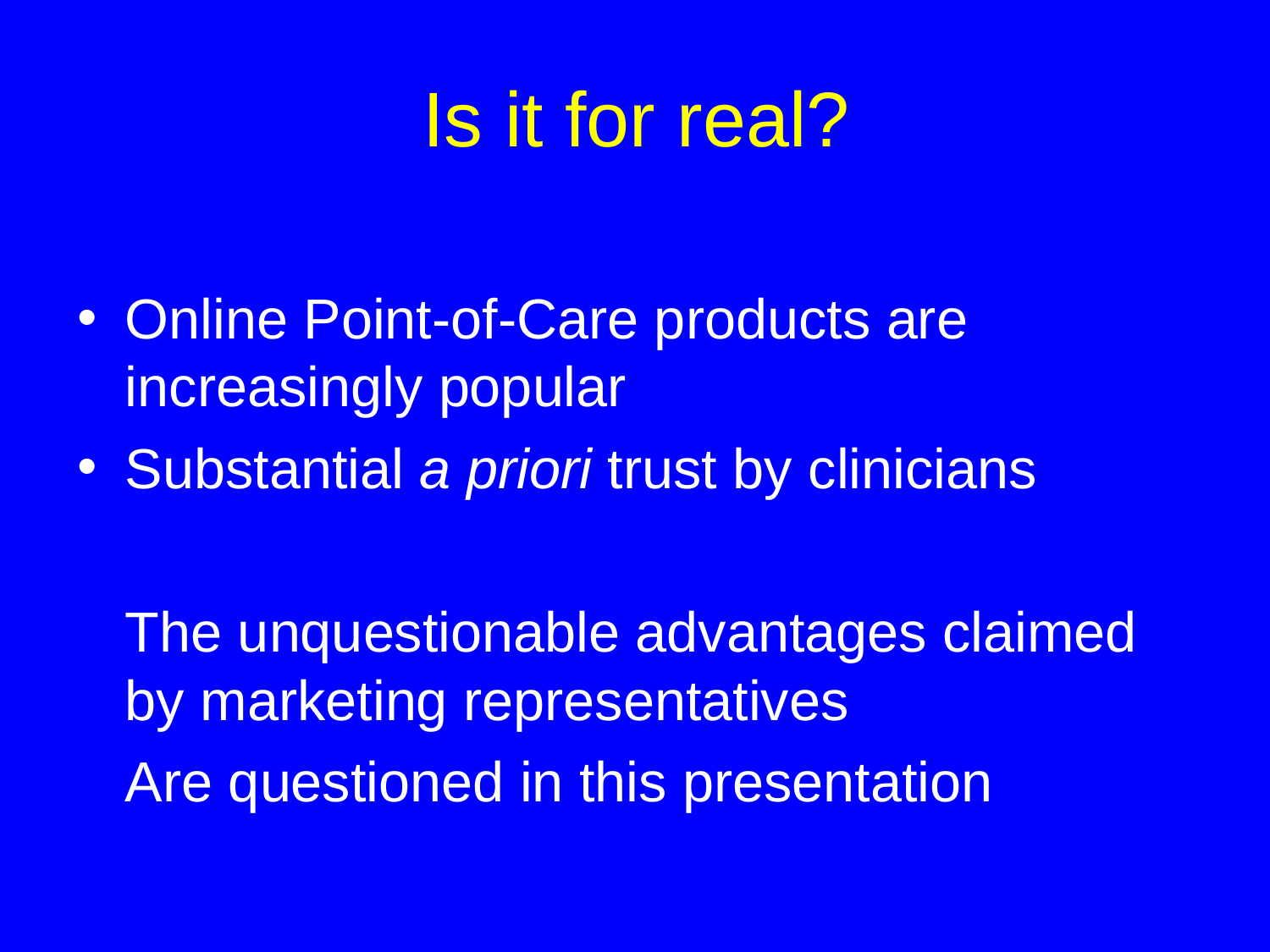

Is it for real?
# Online Point-of-Care products are increasingly popular
Substantial a priori trust by clinicians
The unquestionable advantages claimed by marketing representatives
Are questioned in this presentation

## Slide 6
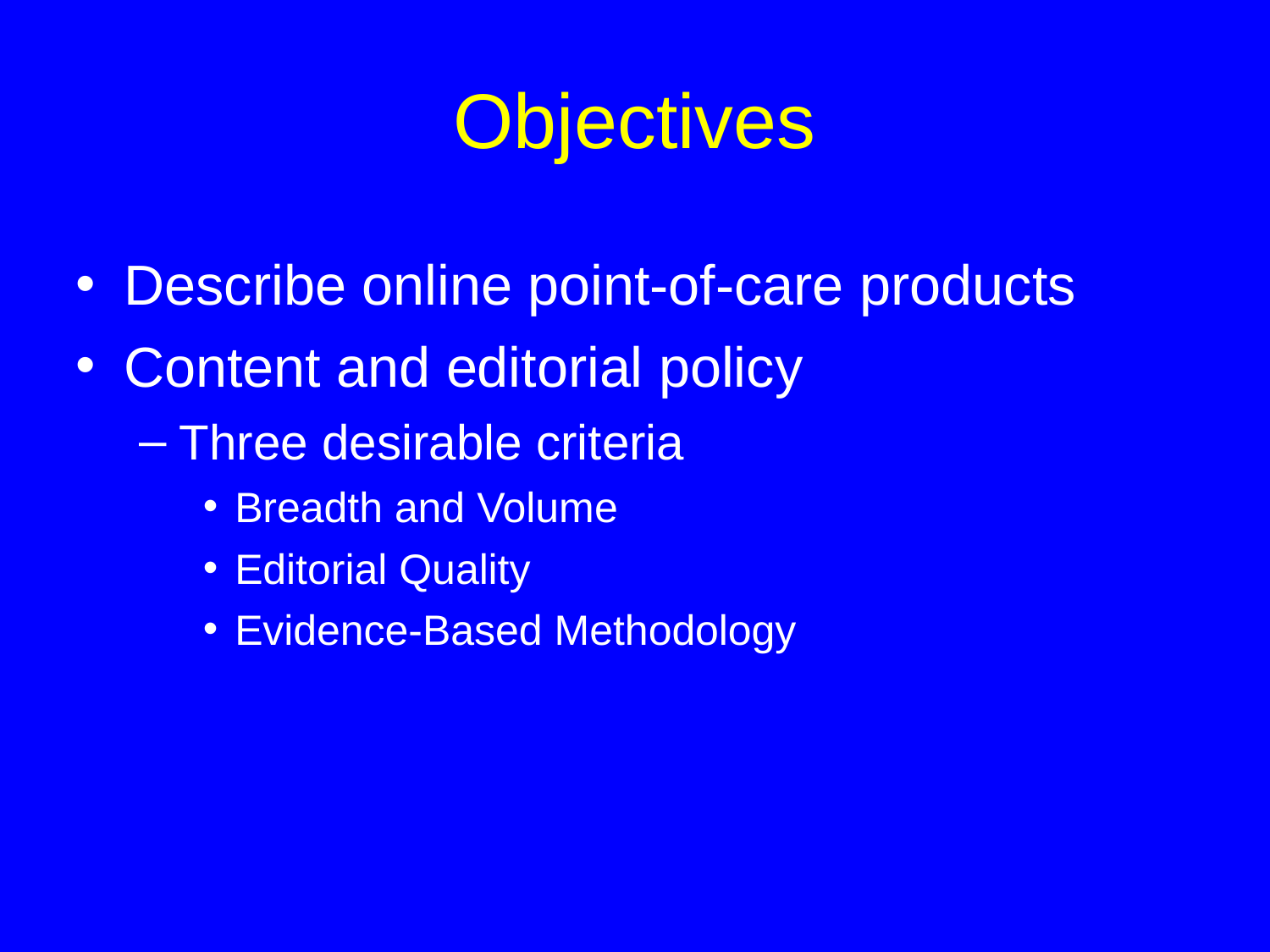

# Objectives
Describe online point-of-care products
Content and editorial policy
Three desirable criteria
Breadth and Volume
Editorial Quality
Evidence-Based Methodology

## Slide 7
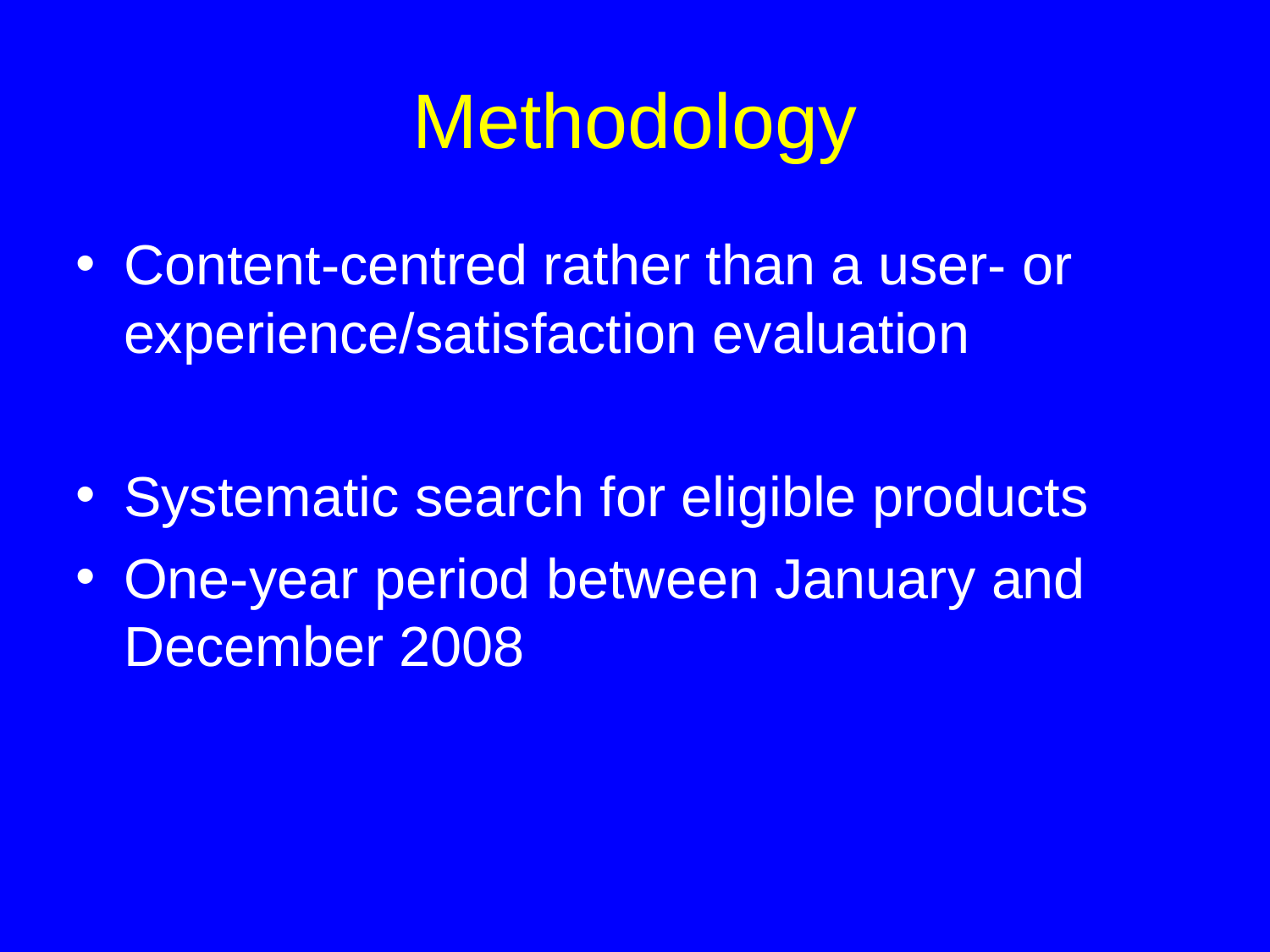

# Methodology
Content-centred rather than a user- or experience/satisfaction evaluation
Systematic search for eligible products
One-year period between January and December 2008

## Slide 8
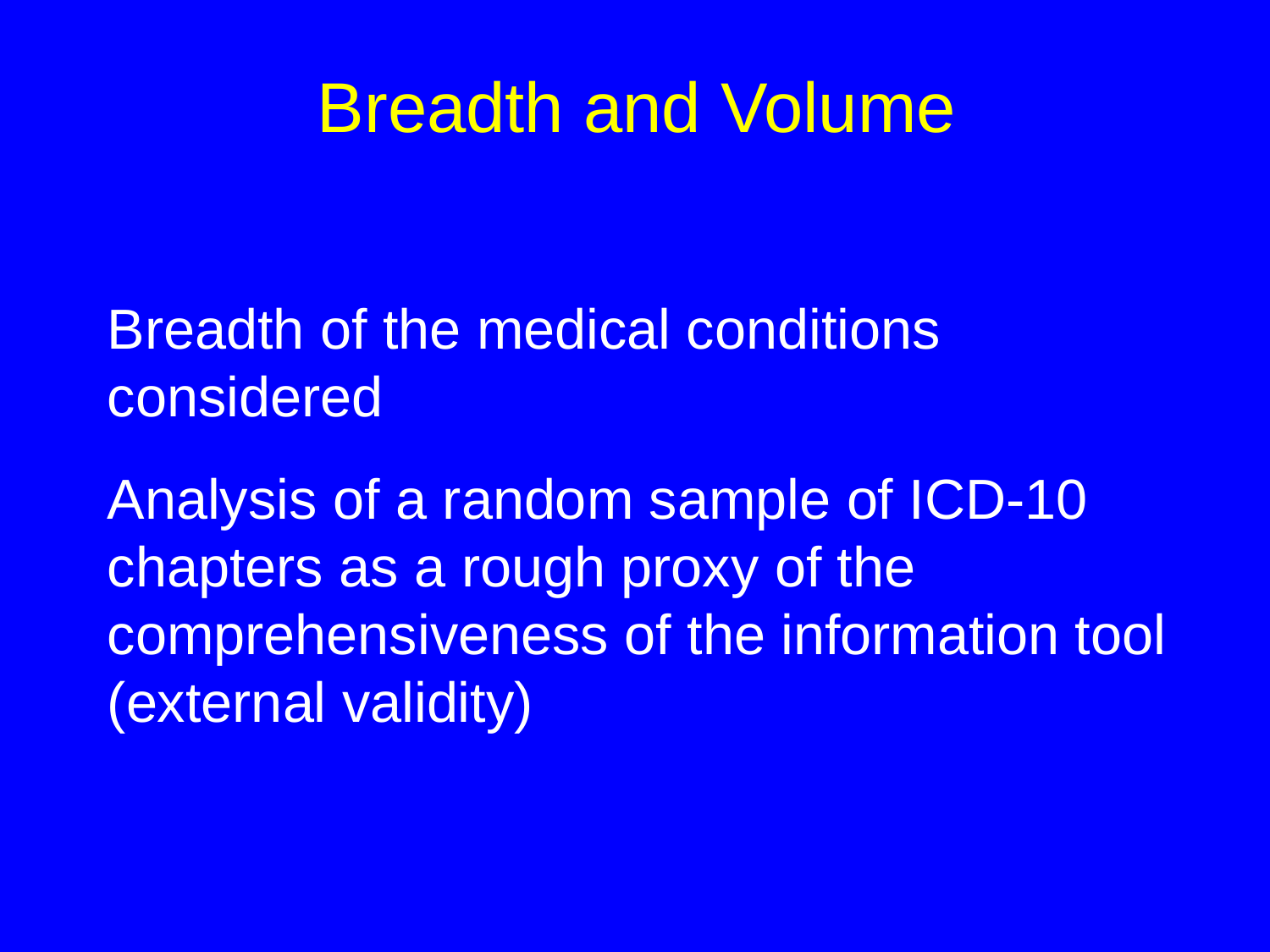

# Breadth and Volume
Breadth of the medical conditions considered
Analysis of a random sample of ICD-10 chapters as a rough proxy of the comprehensiveness of the information tool (external validity)

## Slide 9
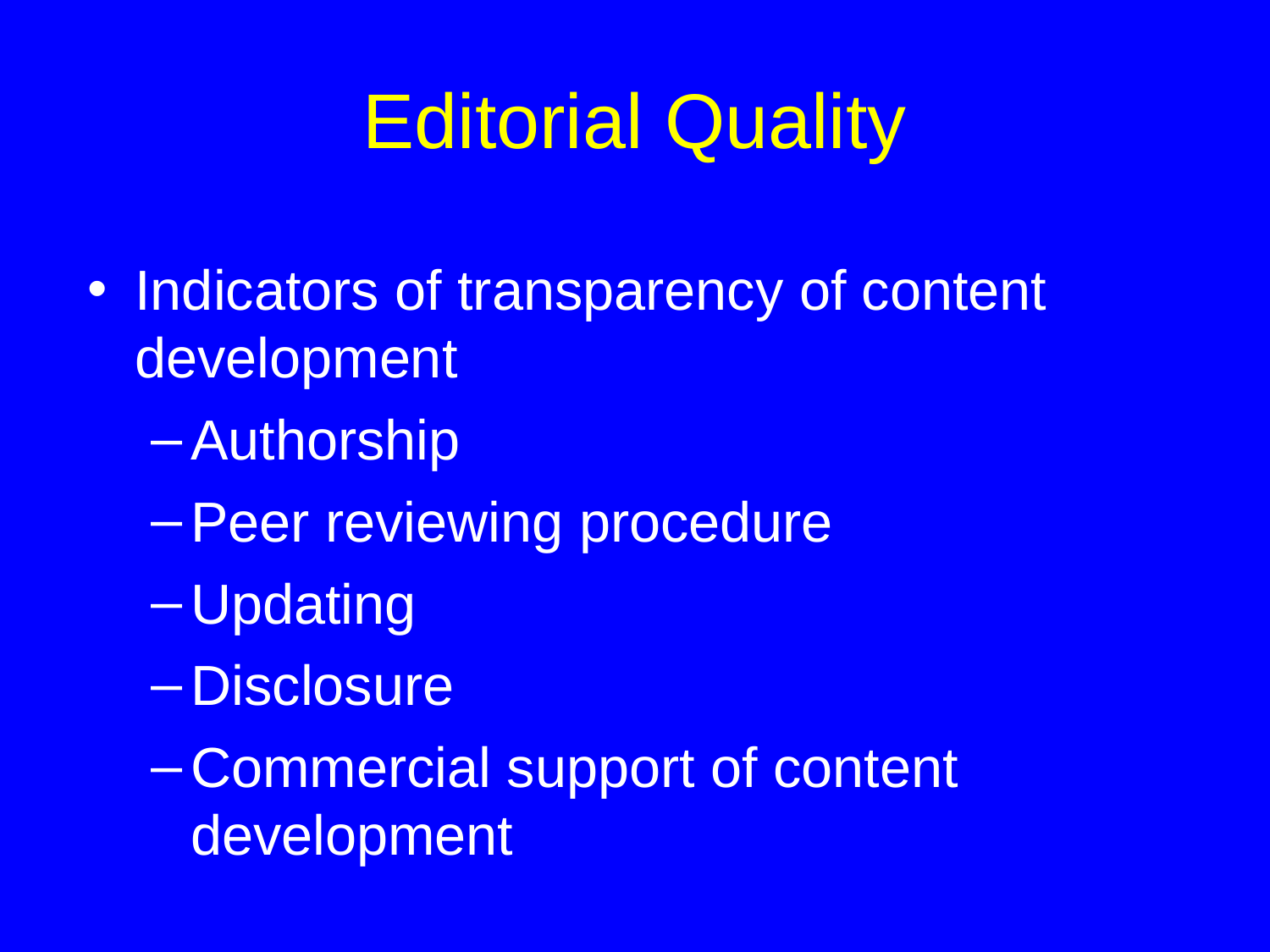

# Editorial Quality
Indicators of transparency of content development
Authorship
Peer reviewing procedure
Updating
Disclosure
Commercial support of content development

## Slide 10
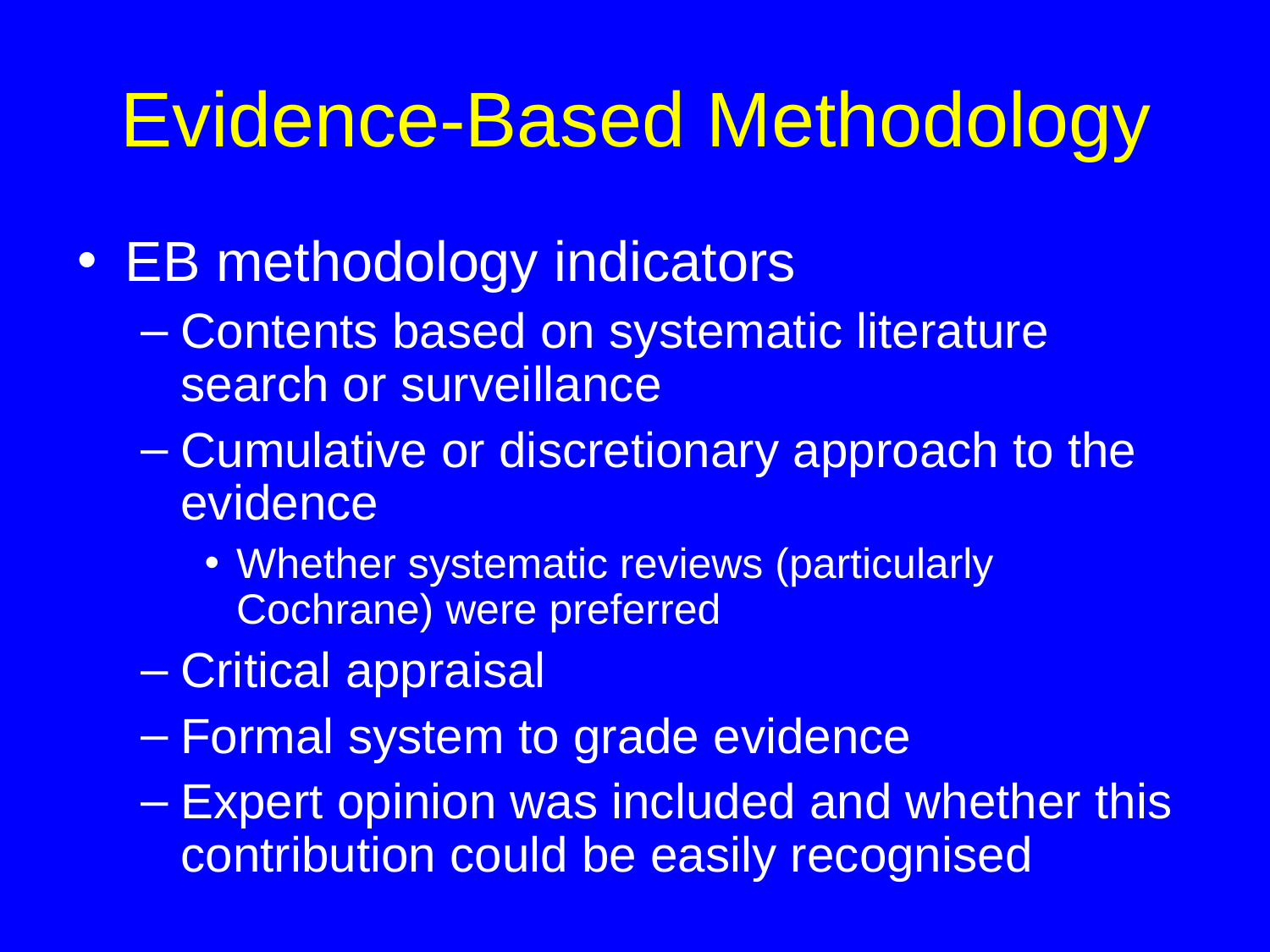

# Evidence-Based Methodology
EB methodology indicators
Contents based on systematic literature search or surveillance
Cumulative or discretionary approach to the evidence
Whether systematic reviews (particularly Cochrane) were preferred
Critical appraisal
Formal system to grade evidence
Expert opinion was included and whether this contribution could be easily recognised

## Slide 11
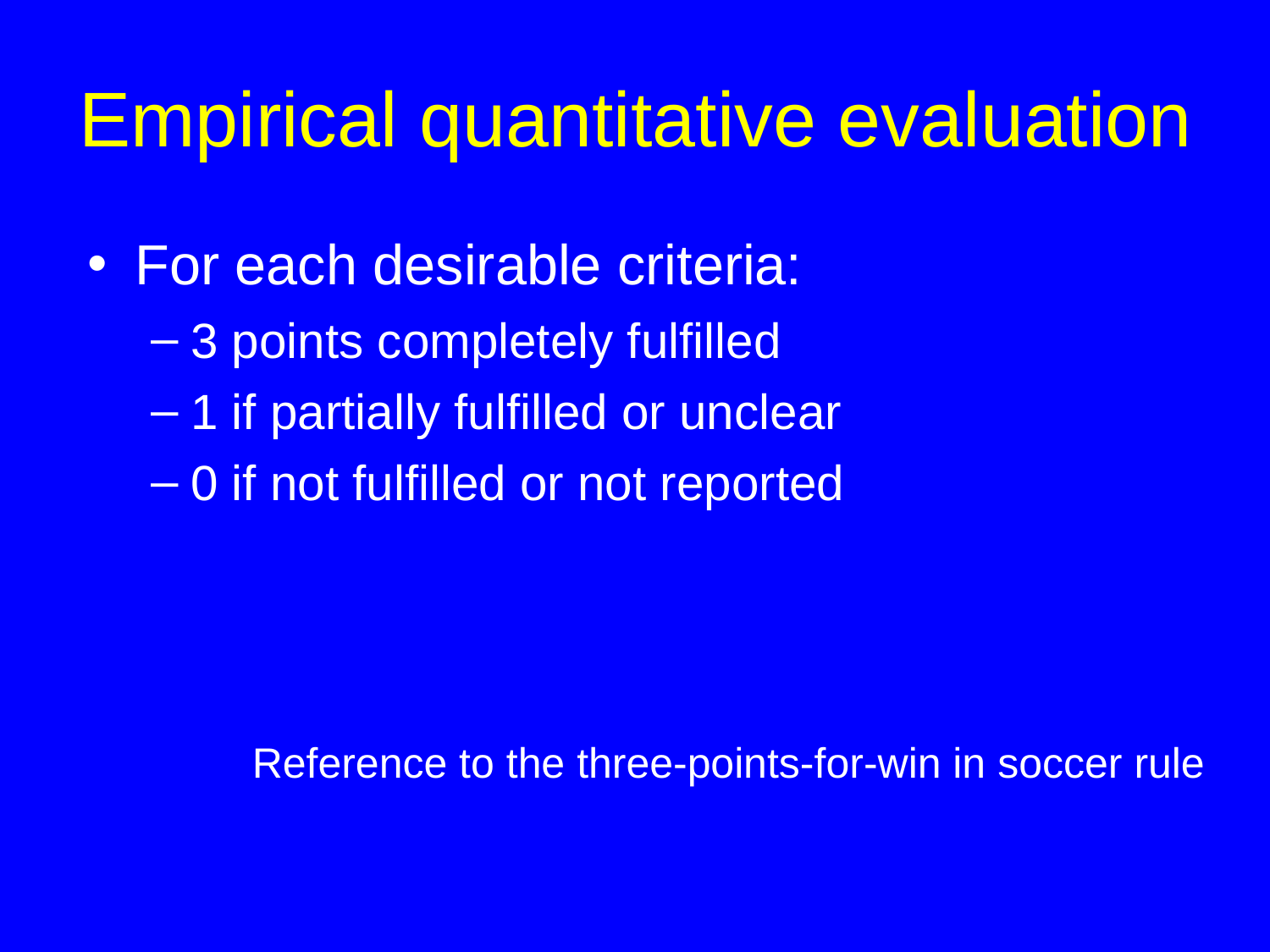

# Empirical quantitative evaluation
For each desirable criteria:
3 points completely fulfilled
1 if partially fulfilled or unclear
0 if not fulfilled or not reported
Reference to the three-points-for-win in soccer rule

## Slide 12
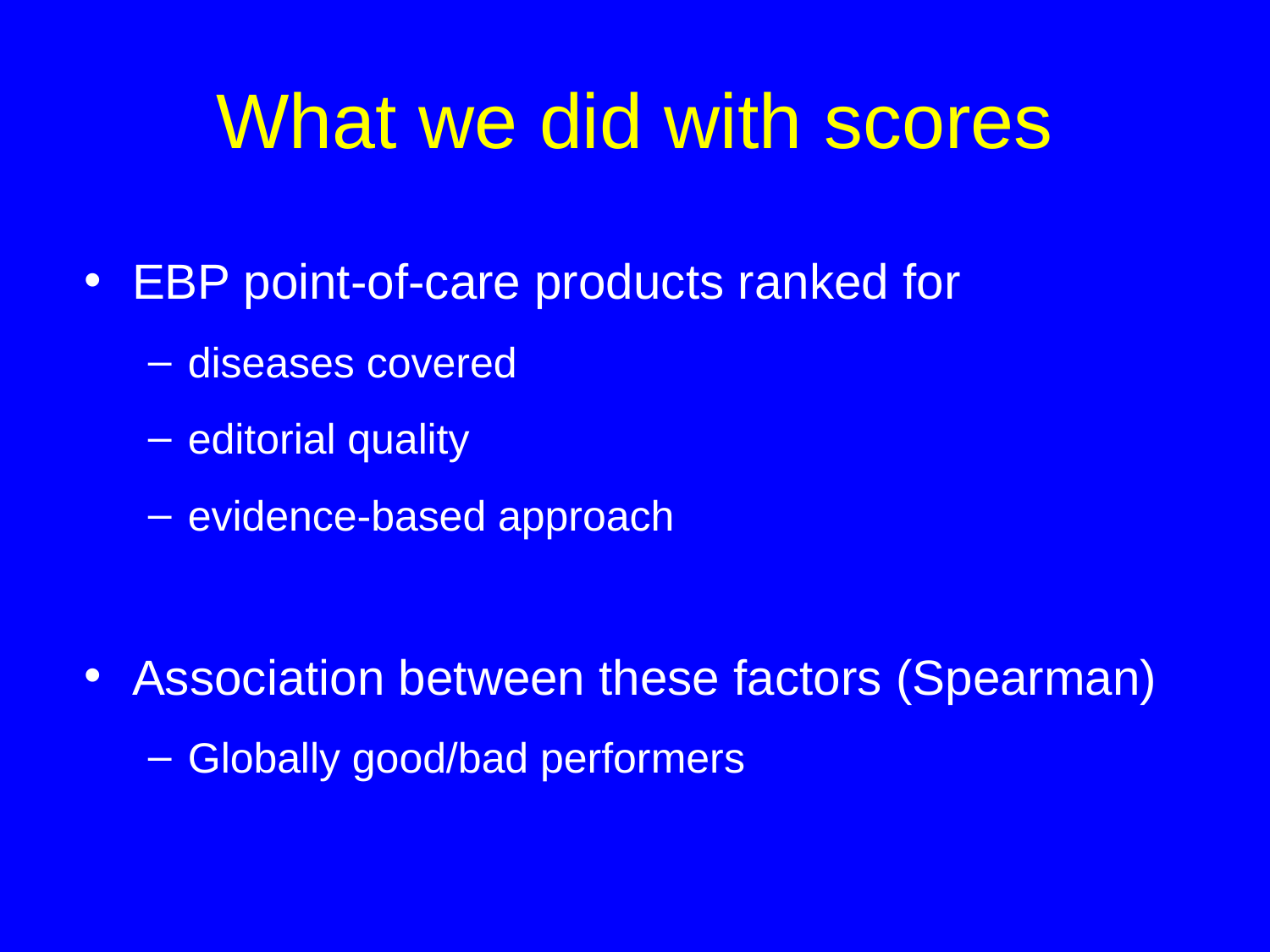

# What we did with scores
EBP point-of-care products ranked for
diseases covered
editorial quality
evidence-based approach
Association between these factors (Spearman)
Globally good/bad performers

## Slide 13
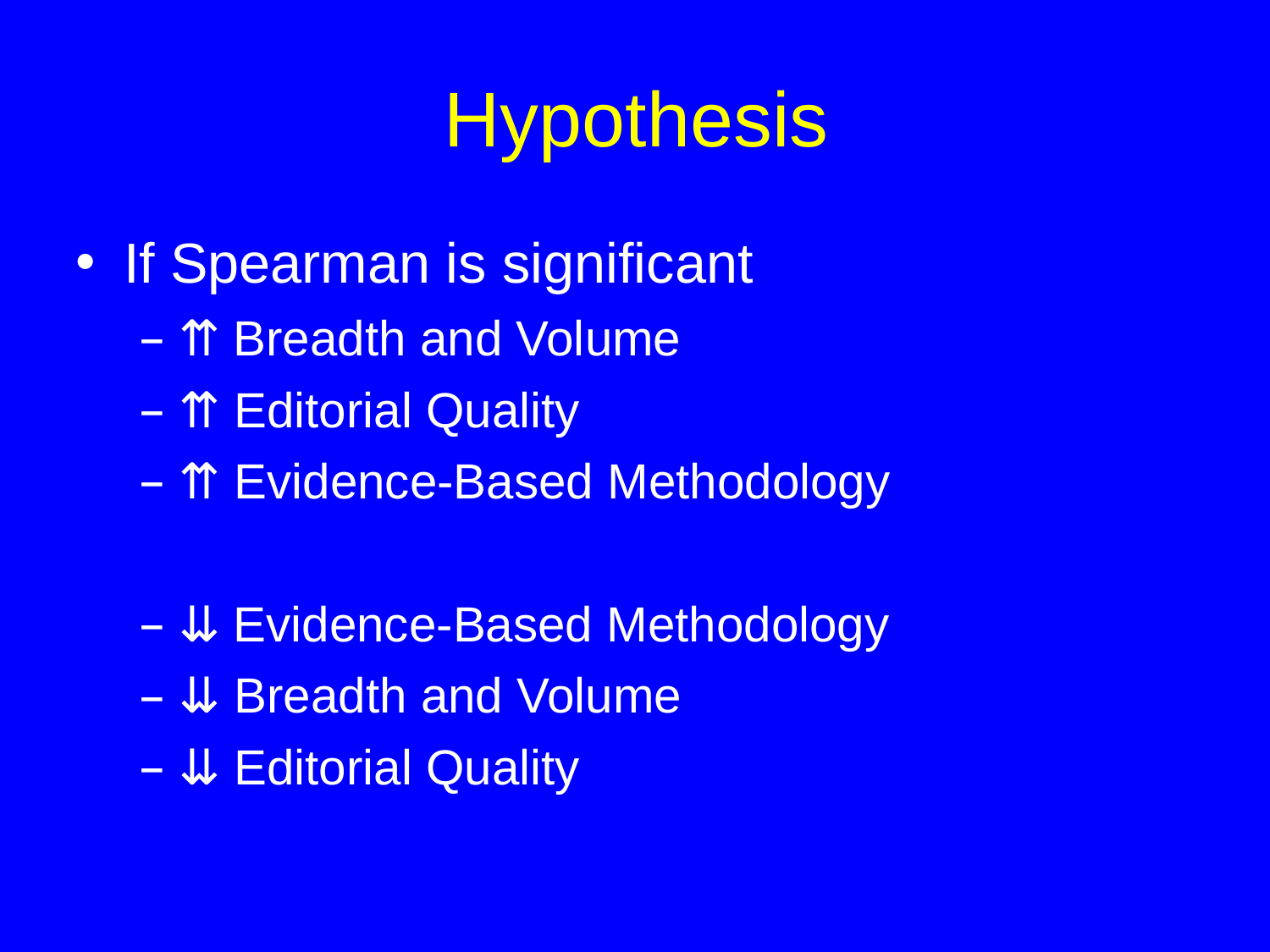

# Hypothesis
If Spearman is significant
⇈ Breadth and Volume
⇈ Editorial Quality
⇈ Evidence-Based Methodology
⇊ Evidence-Based Methodology
⇊ Breadth and Volume
⇊ Editorial Quality

## Slide 14
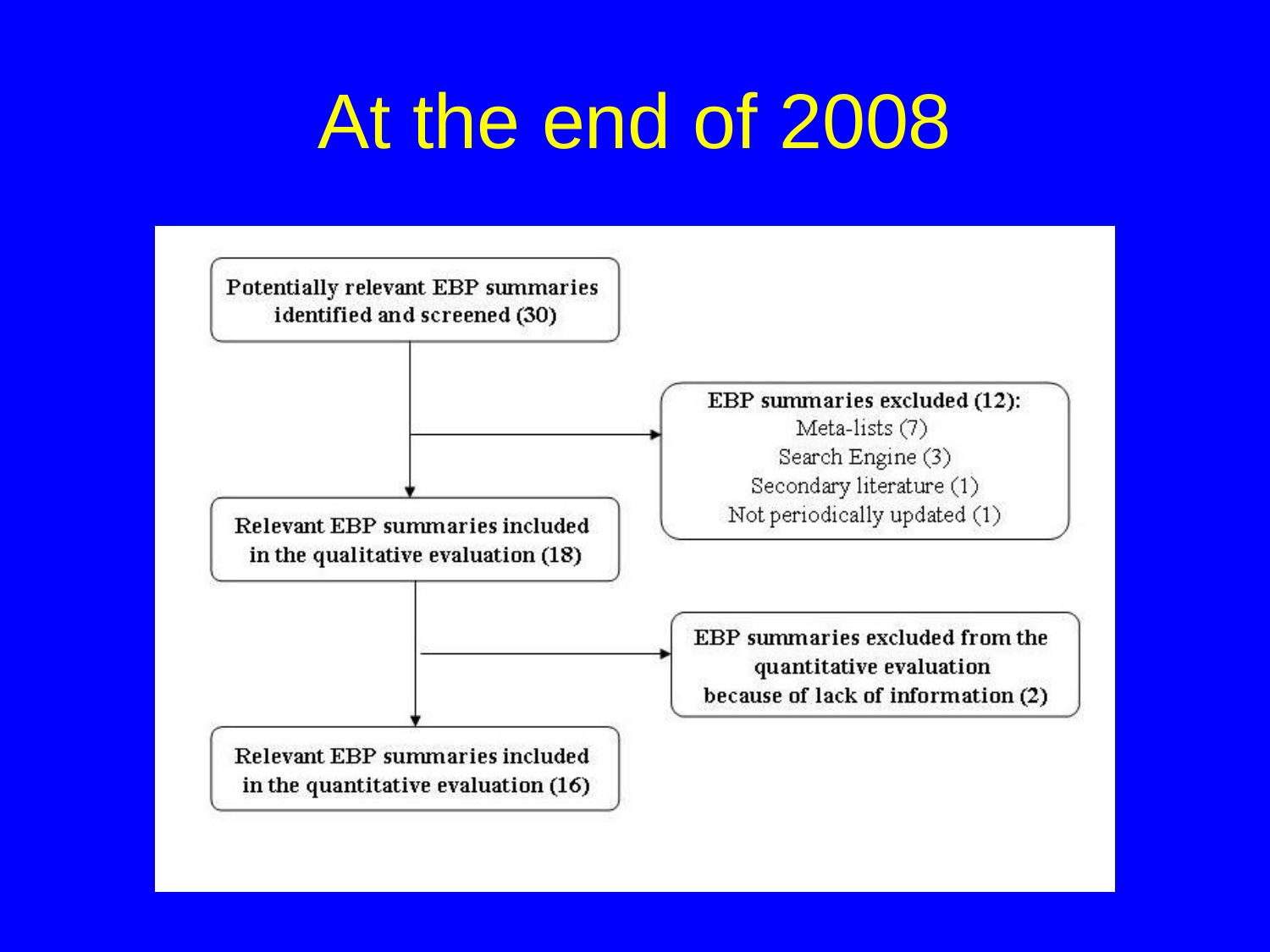

# At the end of 2008

## Slide 15
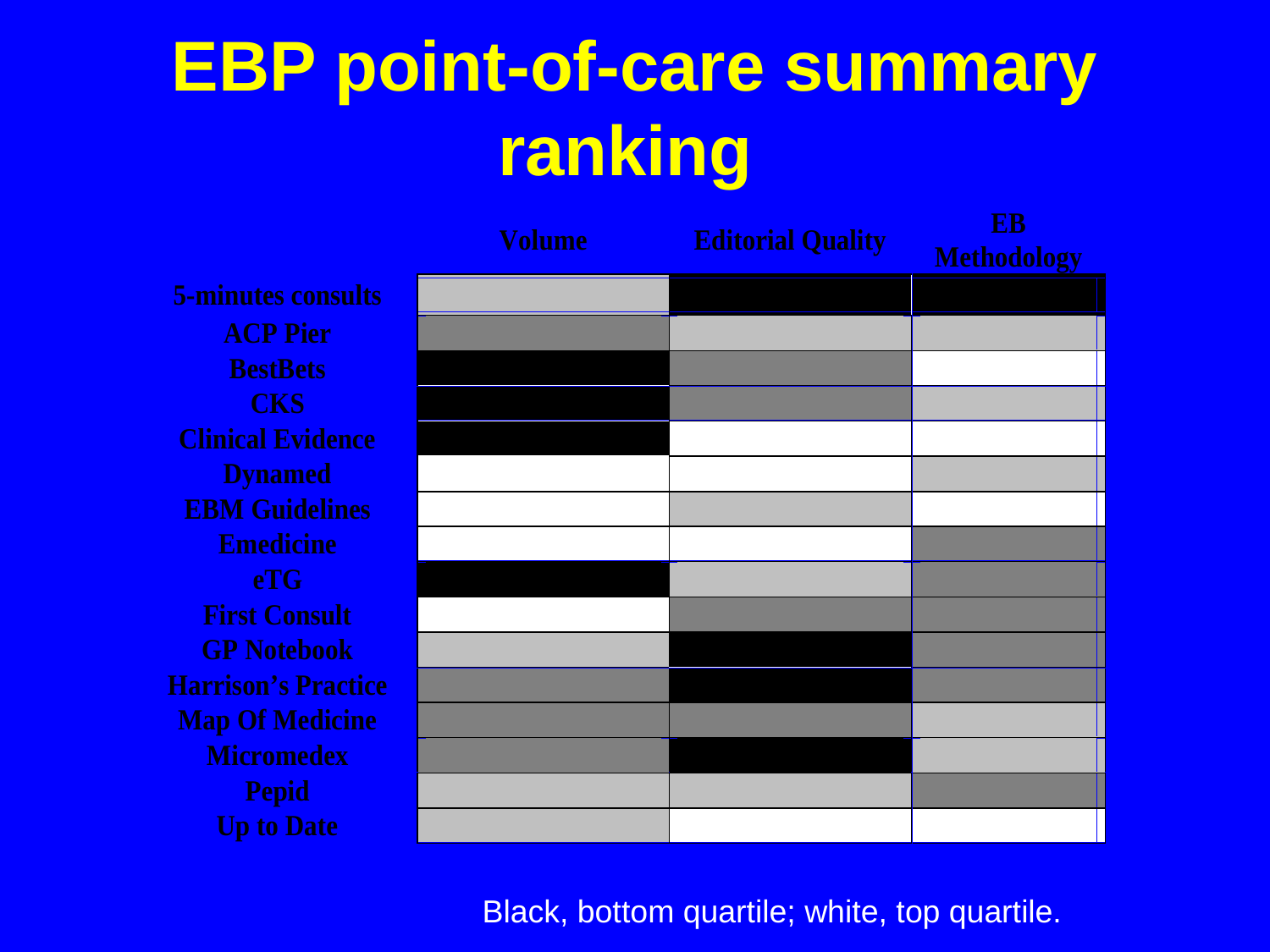

# EBP point-of-care summary ranking
Black, bottom quartile; white, top quartile.

## Slide 16
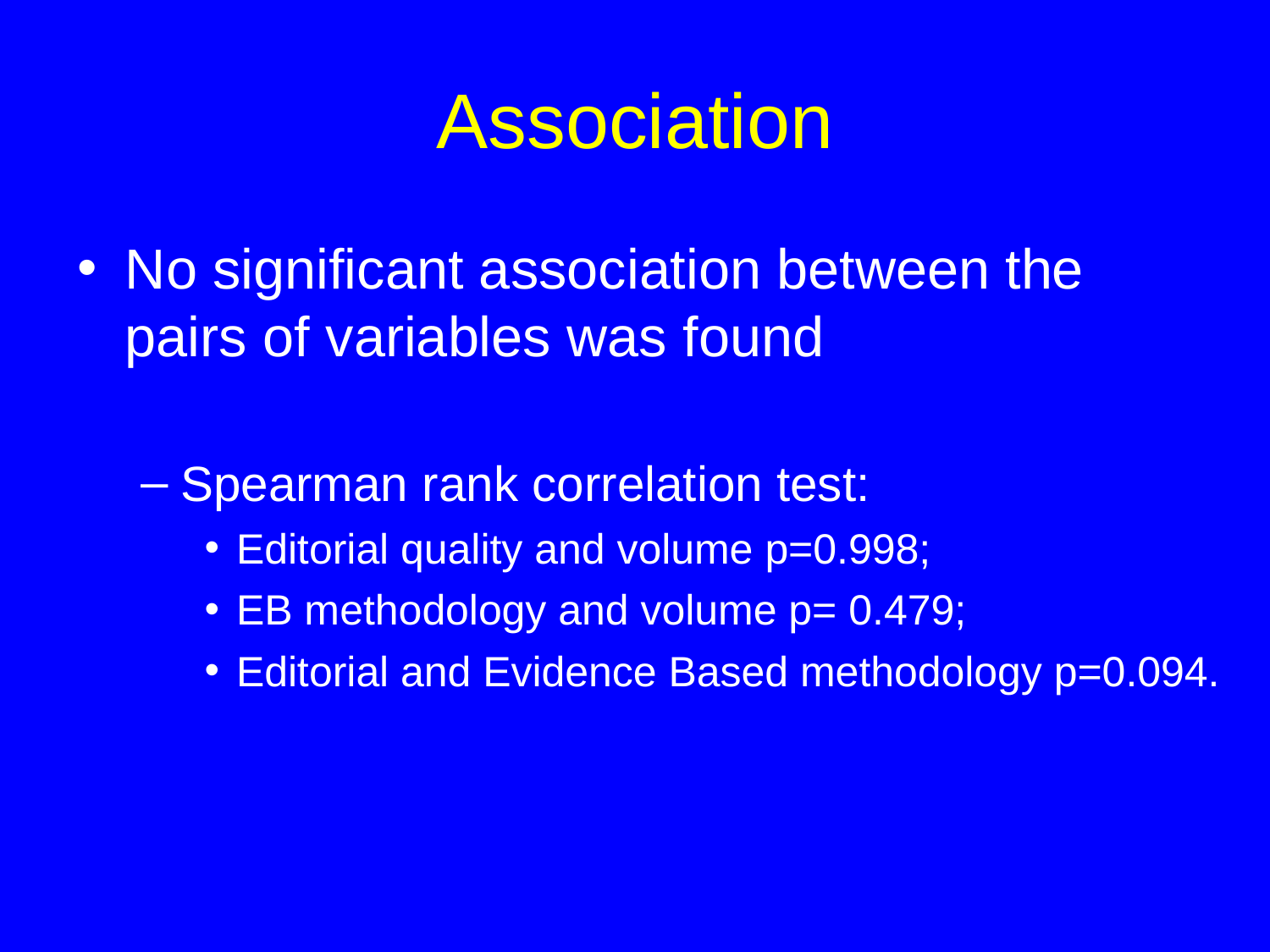

# Association
No significant association between the pairs of variables was found
Spearman rank correlation test:
Editorial quality and volume p=0.998;
EB methodology and volume p= 0.479;
Editorial and Evidence Based methodology p=0.094.

## Slide 17
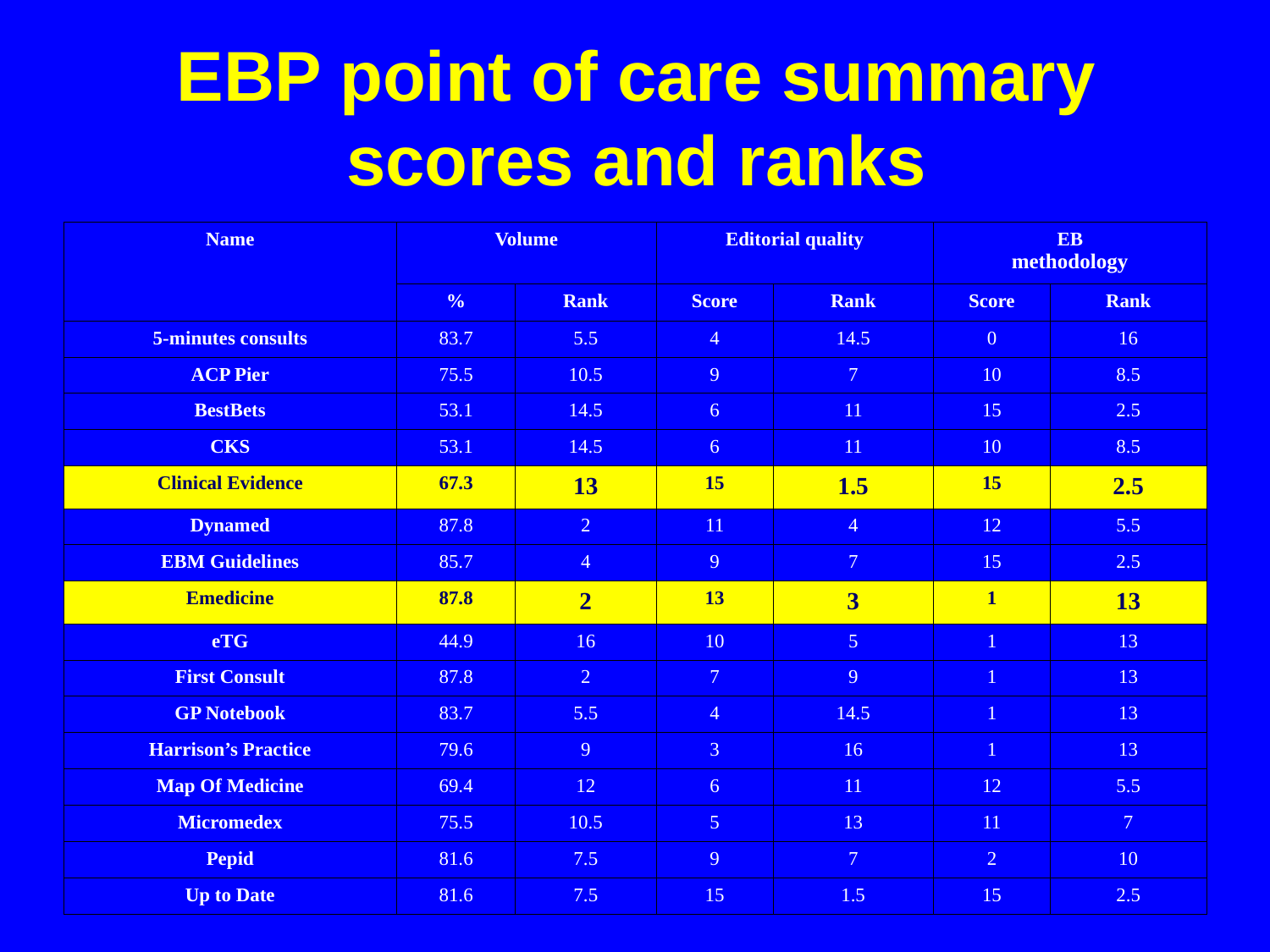

# EBP point of care summary scores and ranks
| Name | Volume | | Editorial quality | | EB methodology | |
| --- | --- | --- | --- | --- | --- | --- |
| | % | Rank | Score | Rank | Score | Rank |
| 5-minutes consults | 83.7 | 5.5 | 4 | 14.5 | 0 | 16 |
| ACP Pier | 75.5 | 10.5 | 9 | 7 | 10 | 8.5 |
| BestBets | 53.1 | 14.5 | 6 | 11 | 15 | 2.5 |
| CKS | 53.1 | 14.5 | 6 | 11 | 10 | 8.5 |
| Clinical Evidence | 67.3 | 13 | 15 | 1.5 | 15 | 2.5 |
| Dynamed | 87.8 | 2 | 11 | 4 | 12 | 5.5 |
| EBM Guidelines | 85.7 | 4 | 9 | 7 | 15 | 2.5 |
| Emedicine | 87.8 | 2 | 13 | 3 | 1 | 13 |
| eTG | 44.9 | 16 | 10 | 5 | 1 | 13 |
| First Consult | 87.8 | 2 | 7 | 9 | 1 | 13 |
| GP Notebook | 83.7 | 5.5 | 4 | 14.5 | 1 | 13 |
| Harrison’s Practice | 79.6 | 9 | 3 | 16 | 1 | 13 |
| Map Of Medicine | 69.4 | 12 | 6 | 11 | 12 | 5.5 |
| Micromedex | 75.5 | 10.5 | 5 | 13 | 11 | 7 |
| Pepid | 81.6 | 7.5 | 9 | 7 | 2 | 10 |
| Up to Date | 81.6 | 7.5 | 15 | 1.5 | 15 | 2.5 |

## Slide 18
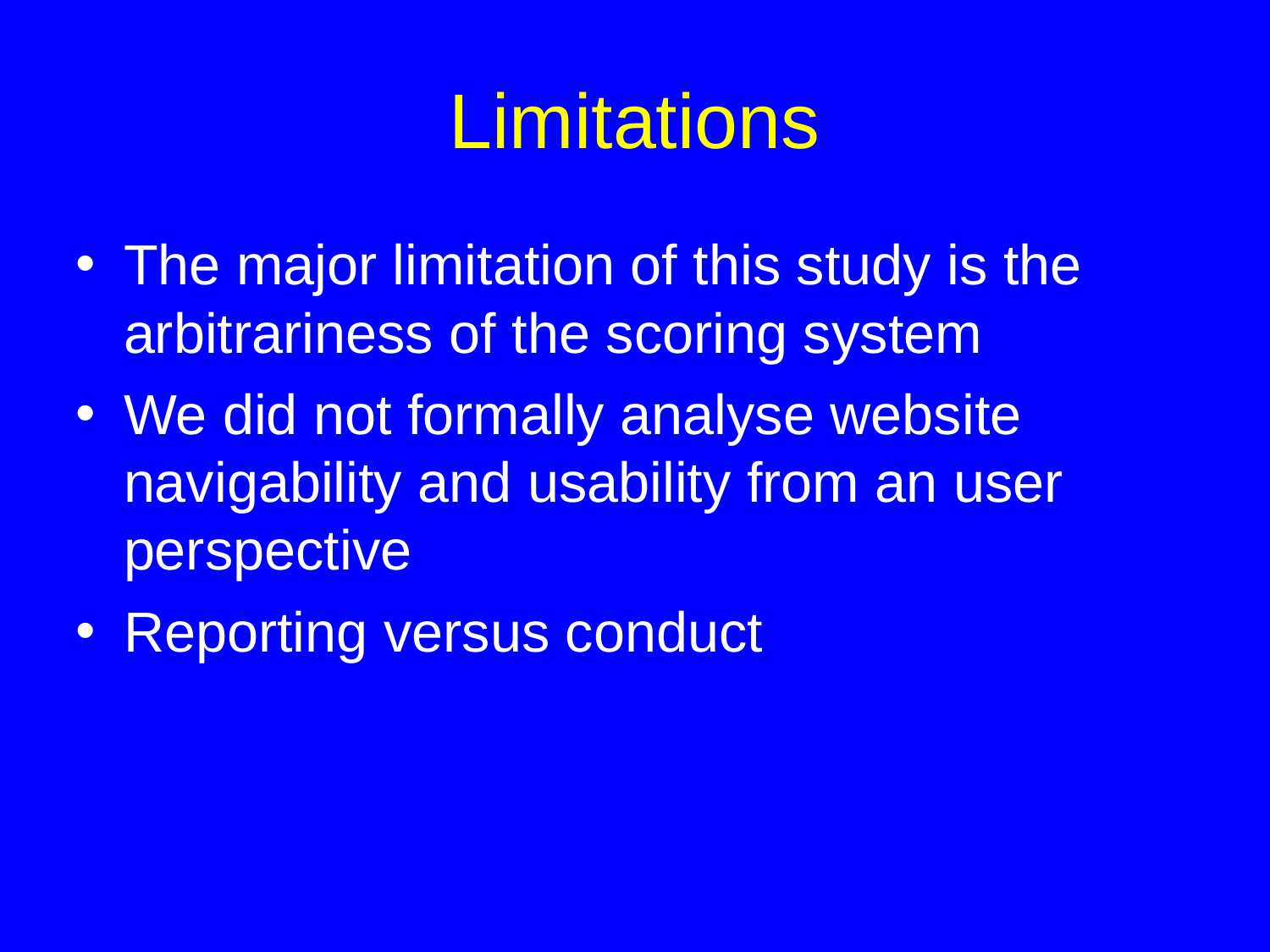

# Limitations
The major limitation of this study is the arbitrariness of the scoring system
We did not formally analyse website navigability and usability from an user perspective
Reporting versus conduct

## Slide 19
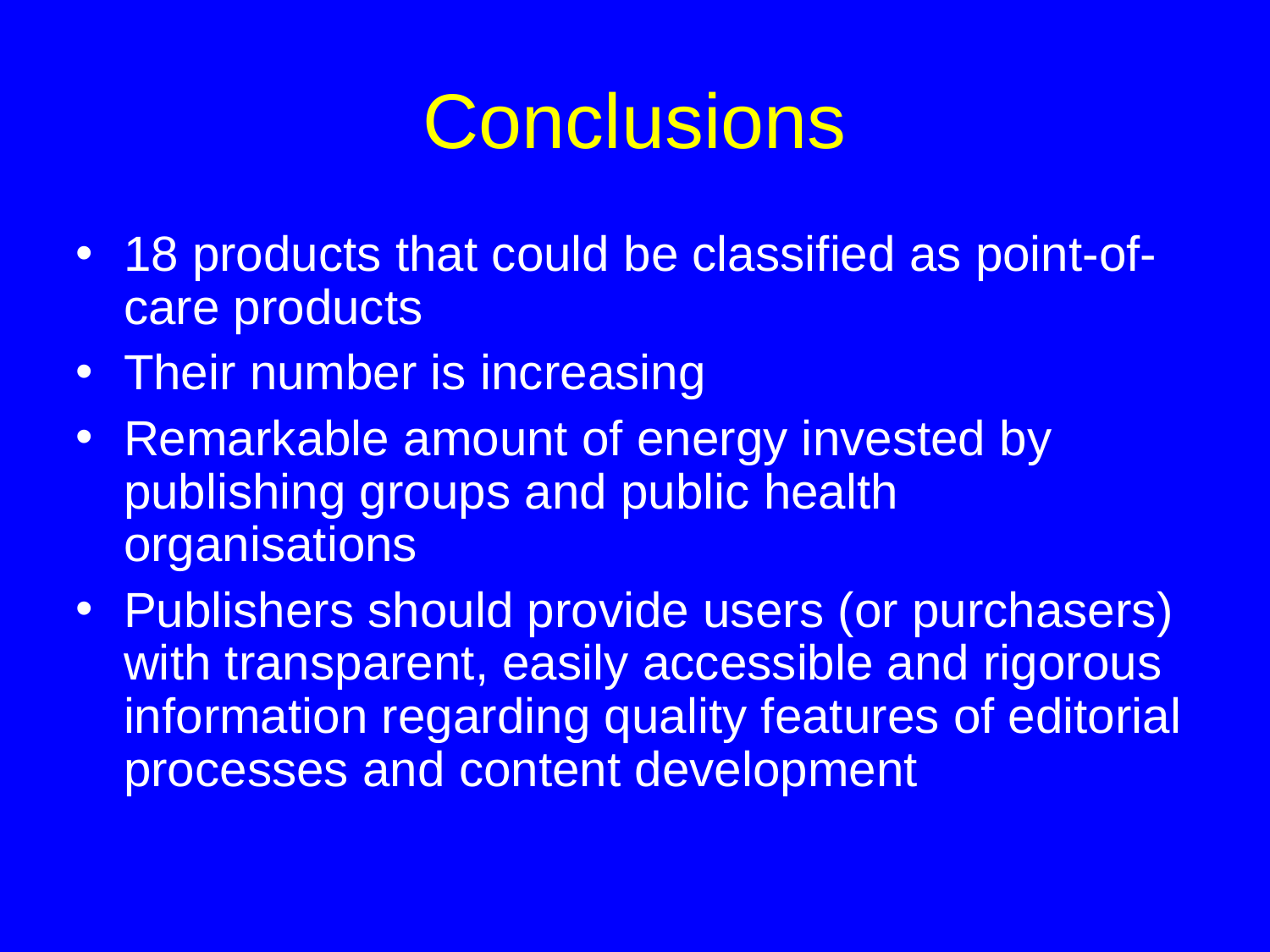

# Conclusions
18 products that could be classified as point-of-care products
Their number is increasing
Remarkable amount of energy invested by publishing groups and public health organisations
Publishers should provide users (or purchasers) with transparent, easily accessible and rigorous information regarding quality features of editorial processes and content development

## Slide 20
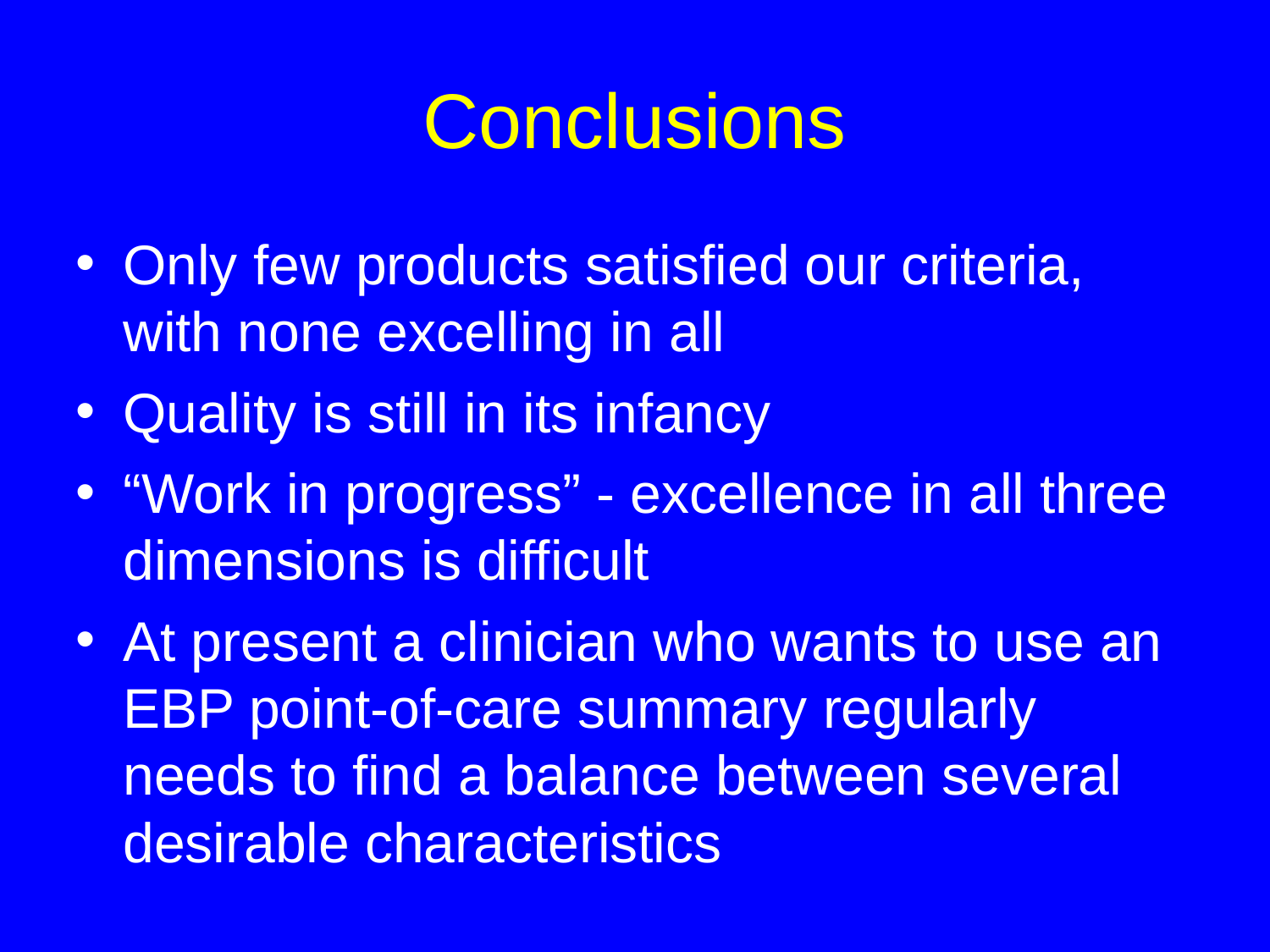

# Conclusions
Only few products satisfied our criteria, with none excelling in all
Quality is still in its infancy
“Work in progress” - excellence in all three dimensions is difficult
At present a clinician who wants to use an EBP point-of-care summary regularly needs to find a balance between several desirable characteristics

## Slide 21
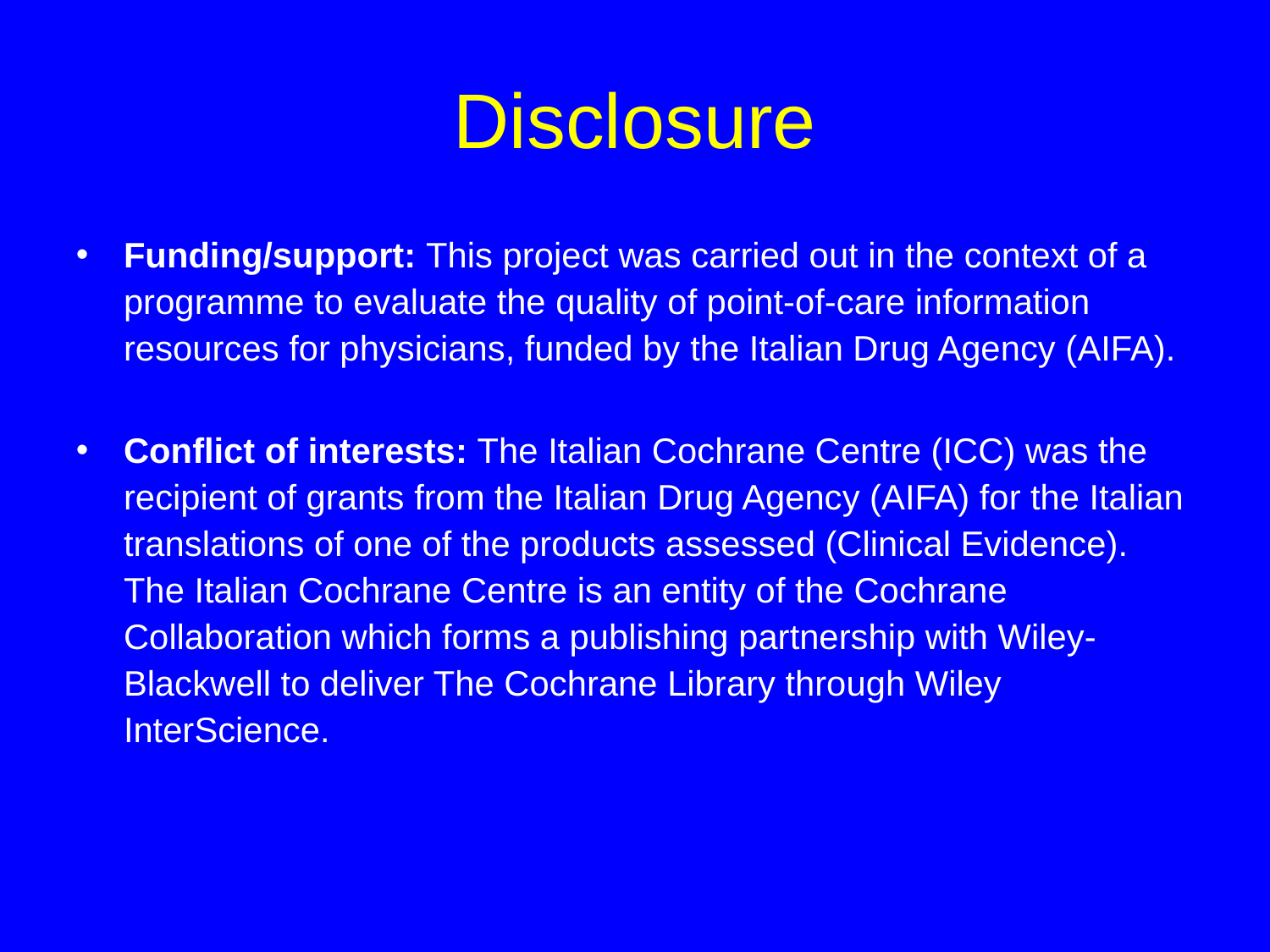

# Disclosure
Funding/support: This project was carried out in the context of a programme to evaluate the quality of point-of-care information resources for physicians, funded by the Italian Drug Agency (AIFA).
Conflict of interests: The Italian Cochrane Centre (ICC) was the recipient of grants from the Italian Drug Agency (AIFA) for the Italian translations of one of the products assessed (Clinical Evidence). The Italian Cochrane Centre is an entity of the Cochrane Collaboration which forms a publishing partnership with Wiley-Blackwell to deliver The Cochrane Library through Wiley InterScience.

## Slide 22
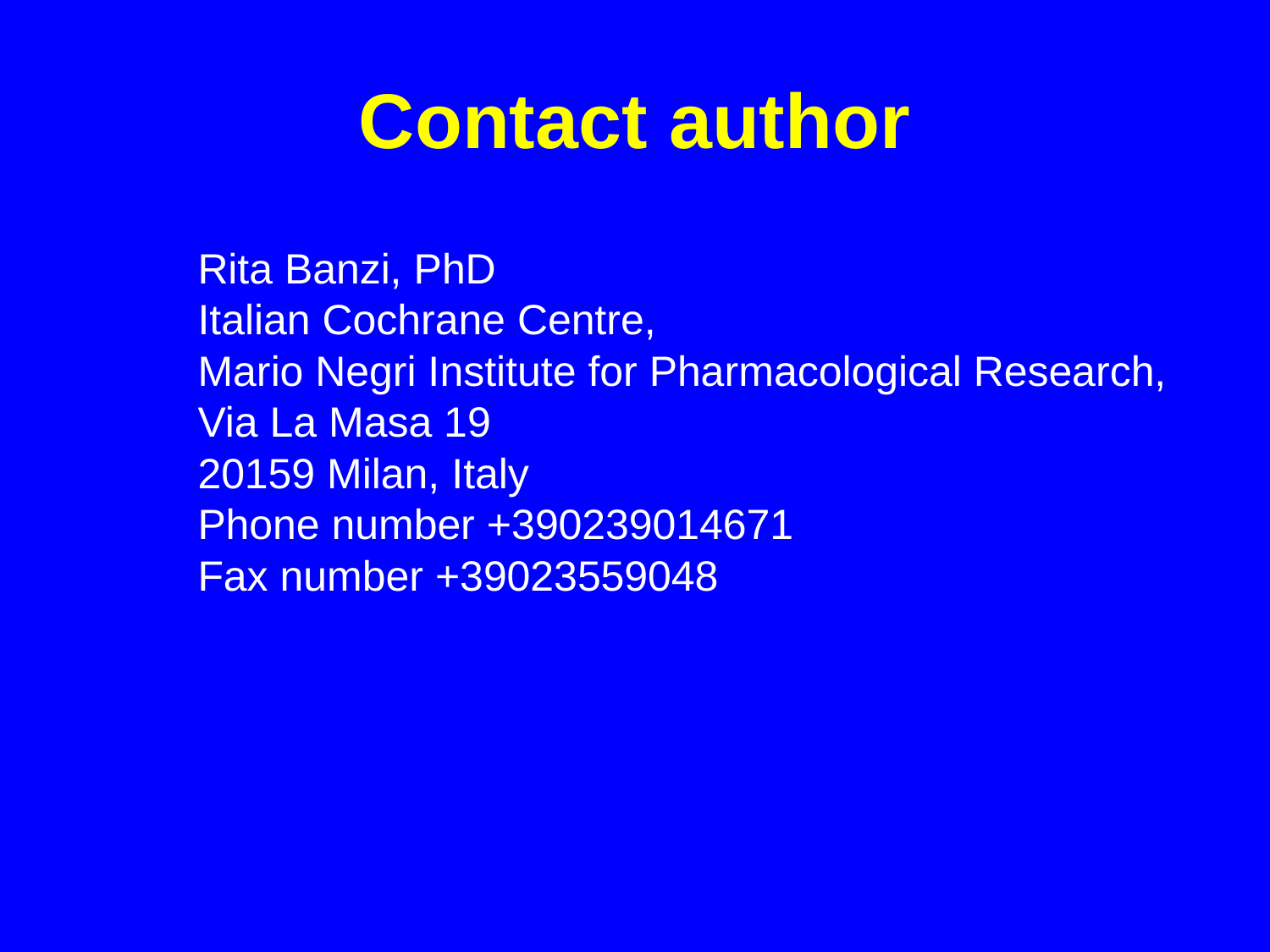

# Contact author
Rita Banzi, PhD
Italian Cochrane Centre,
Mario Negri Institute for Pharmacological Research,
Via La Masa 19
20159 Milan, Italy
Phone number +390239014671
Fax number +39023559048
banzi@marionegri.it
www.cochrane.it
www.marionegri.it
